# Normalized unitary synaptic signaling of the hippocampus and entorhinal cortex predicted by deep learning of experimental recordings

**DOI:** 10.1038/s42003-022-03329-5

**Published:** 2022-05-05

**Authors:** Keivan Moradi, Zainab Aldarraji, Megha Luthra, Grey P. Madison, Giorgio A. Ascoli

**Affiliations:** 1 Interdisciplinary Neuroscience Program and Krasnow Institute for Advanced Study, George Mason University, Fairfax, VA USA; 2 Bioengineering Department and Volgenau School of Engineering, George Mason University, Fairfax, VA USA; 3 Chemistry and Biochemistry Department, College of Science, George Mason University, Fairfax, VA USA; 4 Present Address: Department of Neurobiology, David Geffen School of Medicine, University of California, Los Angeles, CA USA

**Keywords:** Neuroscience, Physiology

## Abstract

Biologically realistic computer simulations of neuronal circuits require systematic data-driven modeling of neuron type-specific synaptic activity. However, limited experimental yield, heterogeneous recordings conditions, and ambiguous neuronal identification have so far prevented the consistent characterization of synaptic signals for all connections of any neural system. We introduce a strategy to overcome these challenges and report a comprehensive synaptic quantification among all known neuron types of the hippocampal-entorhinal network. First, we reconstructed >2600 synaptic traces from ∼1200 publications into a unified computational representation of synaptic dynamics. We then trained a deep learning architecture with the resulting parameters, each annotated with detailed metadata such as recording method, solutions, and temperature. The model learned to predict the synaptic properties of all 3,120 circuit connections in arbitrary conditions with accuracy approaching the intrinsic experimental variability. Analysis of data normalized and completed with the deep learning model revealed that synaptic signals are controlled by few latent variables associated with specific molecular markers and interrelating conductance, decay time constant, and short-term plasticity. We freely release the tools and full dataset of unitary synaptic values in 32 covariate settings. Normalized synaptic data can be used in brain simulations, and to predict and test experimental hypothesis.

## Abstract (web summary)

A deep learning model trained on roughly 2,600 synaptic traces from hippocampal electrophysiology datasets demonstrates how specific covariates influence synaptic signals.

## Introduction

The discovery of place cells and grid cells underscored the importance of the hippocampal formation as a key neural substrate for spatial navigation^1,2^, fueling an intensive investigation of this brain region. Understanding spatial coding requires a model of the information flow in the underlying cellular circuit. Synapses mediate neuronal communication by enabling the transmission of signal from the axon of a (sender) neuron to the dendrite or perisomatic area of a (receiver) neuron. An electrical signal is thus recordable from the postsynaptic cell upon activation of the presynaptic cell. Different synapses produce distinct signals ultimately orchestrating behavior and cognition^3^. For example, the release probability, conductance, and short-term plasticity (ST-P) vary among pairs of neuron types^4^. Plastic changes in synaptic signaling subserve adaptive processes underlying memory. Identifying aberrant synaptic dynamics is crucial to the elucidation of the pathophysiology of diseases such as schizophrenia and depression^5,6^. Yet, the synaptic physiology of most neuronal connections remains poorly understood.

The summed synaptic activity of multiple contacts connecting two neurons is a *unitary* signal. Unitary synaptic signals are typically measured by paired recording^7^, allowing for post-hoc identification of both presynaptic and postsynaptic neuronal types. Unfortunately, paired recordings are based on blind probing with a low success rate in finding connected pairs. Accordingly, sample sizes for this method are typically small. Collating recordings from different studies may increase statistical power if they can be mapped to a common framework. Such a framework needs to unify synaptic measurement methods (synaptometrics), experimental conditions such as temperature and slicing preparation methods, and classification of neuronal types.

The knowledge base Hippocampome.org provides a useful starting point by identifying 122 neuron types based on their main neurotransmitter (glutamate or GABA), their dendritic and axonal morphologies, and their molecular expression. Since neuronal connections require the anatomical co-location of a presynaptic axon and a postsynaptic dendrite (or soma), synapses could be classified based on the morphological patterns of the corresponding neurons^8^. Specifically, if a neuron type sends its axons to an anatomical subregion and layer in which another neuron type extends its dendrites, these two neurons can make a connection. The set of all axonal-dendritic co-locations is then trimmed by excluding experimentally refuted connectivity to yield a list of potential connections. Thus, anatomical constraints and known connection specificities are used to reduce the number of potential connections from all 14,884 (122 × 122) pairs of neuron types to only 3,120 (~21%) in the rodent hippocampus and entorhinal cortex^9,10^. Nevertheless, Hippocampome.org lacked until now a quantitative description of normalized synaptic physiology for all potential connections in the circuit.

To coalesce synaptic physiology data from the hippocampal formation, we mined approximately 1,200 publications, annotated more than 2,600 synaptic electrophysiology traces or values, extracted the synaptometrics, annotated the recording methods and experimental conditions, and mapped the data to the neuron types and potential connections of Hippocampome.org^11^. However, the data are in various formats, requiring unification into a common formalism. This can be achieved using a phenomenological description of synaptic dynamics that summarizes synaptic properties in a low dimensional parametrization of the ground truth^12–14^. In such an approach, synaptic amplitude is defined by a conductance (g), decay kinetics with a deactivation time constant (*τ*_d_), and short-term plasticity (ST-P) through the dynamics of synaptic resource utilization and recovery determined by three parameters: a recovery time constant (*τ*_r_), a facilitation time constant (*τ*_f_), and the utilization ratio (U).

Large g values lead to high synaptic amplitudes, and large *τ*_d_ values result in slow kinetics. Depending on the calcium concentration in the presynaptic terminal, each synaptic event increases resource utilization rate, reflecting the number of released neurotransmitters and postsynaptic receptors occupied at any moment. U determines the utilization increment proportion after each event, but it is not the only factor. Resource utilization rate diminishes between events as calcium is reabsorbed. The utilization reduction pace is determined by *τ*_f_. When *τ*_f_ is large, utilization reduction speed is slow, and synapses have a higher probability to facilitate. Since synaptic resources are limited, utilization may cause depletion. Therefore, synapses could have fewer resources for the next event, unless they recover quickly. The factor *τ*_r_ determines the recovery speed. High *τ*_r_ indicates lower recovery rate which makes synapses more likely to undergo short-term depression.

**Fig. 1:**
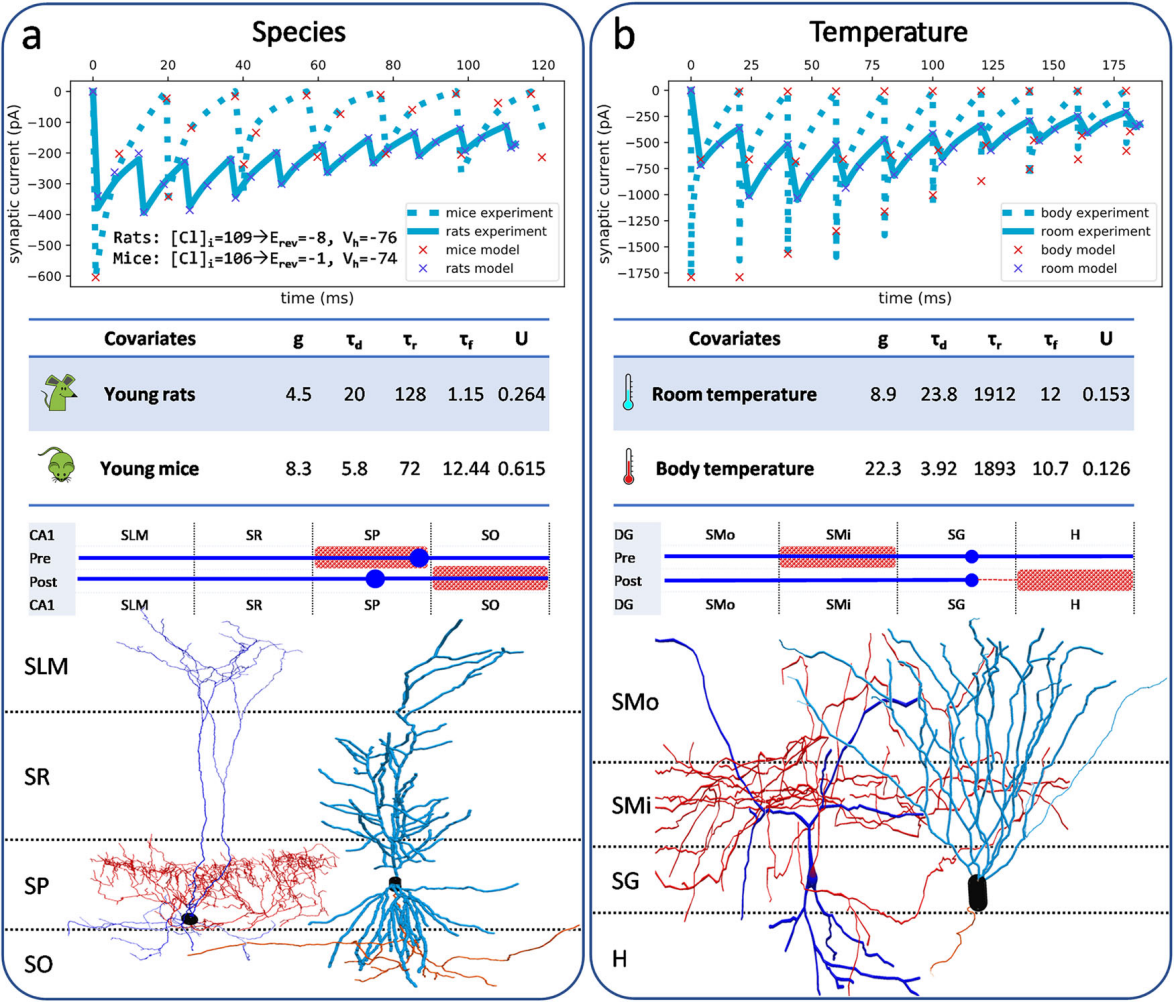
Impact of covariates on synaptic properties. **a** Synaptic traces from two studies^67,68^ recording GABAergic signals from CA1 Axo-axonic to CA1 Pyramidal cells in different species. Differences in intracellular solutions are also indicated. Note large differences in g, *τ*_d,_ and U. Neuronal morphologies are from NeuroMorpho.Org^69–71^ and displayed with schematics based on their axonal (red pattern) and soma-dendritic (blue dot and line) distributions across layers (SMo/SMi, outer/inner molecular; SG, granule; H, hilus; SLM, lacunosum-moleculare; SR, radiatum; SP, pyramidal; SO, oriens). **b** GABAergic signals from DG HICAP to DG Granule cells recorded at two different temperatures^72^. All other covariates were identical. Neuronal morphologies are from NeuroMorpho.Org^69,73,74^.

Quantifying synaptic physiology with the aforementioned parameters enables the unification of diverse experimental data. Nevertheless, different covariates, including species, sex, temperature, and recording modality, make it challenging to compare synapses beyond the scope of the original studies (Fig. 1). Published reports also do not cover all potential connections. Synaptic data in Hippocampome.org are only available for ~84% of potential connections in the hippocampal formation. Moreover, due to the often-ambiguous identification of cell types, each synaptic signal is typically mappable to several potential connections^11^. To solve these problems, the mined data require proper integration^15–17^. Specifically, a comprehensive data model is needed to normalize existing data and infer missing information. Deep learning is a powerful tool for data integration and supports multi-target regression^18–21^. In fact, trial-to-trial heterogeneity may increase the robustness of machine learning^22^. Despite its successes in other fields, deep learning has never been employed to integrate synaptic electrophysiology data.

This study introduces a strategy to normalize unitary synaptic properties and employs it to generalize the available electrophysiology data by inferring the missing information. First, we effectively reconciled data collected through multifarious methods by fitting the quantitative measurements of recorded connections with a parametric synapse model. Then, we trained a predictive deep learning model to normalize the data for covariates and validated the prediction accuracy against the measured experimental variability. The model can infer missing values in arbitrary conditions and resolve ambiguous neuronal identities. Thus, for the first time, we comprehensively analyzed the normalized synaptic properties of all potential connections of the rodent entorhinal-hippocampal network and unraveled crucial factors governing synaptic physiology.

## Results

**Fig. 2:**
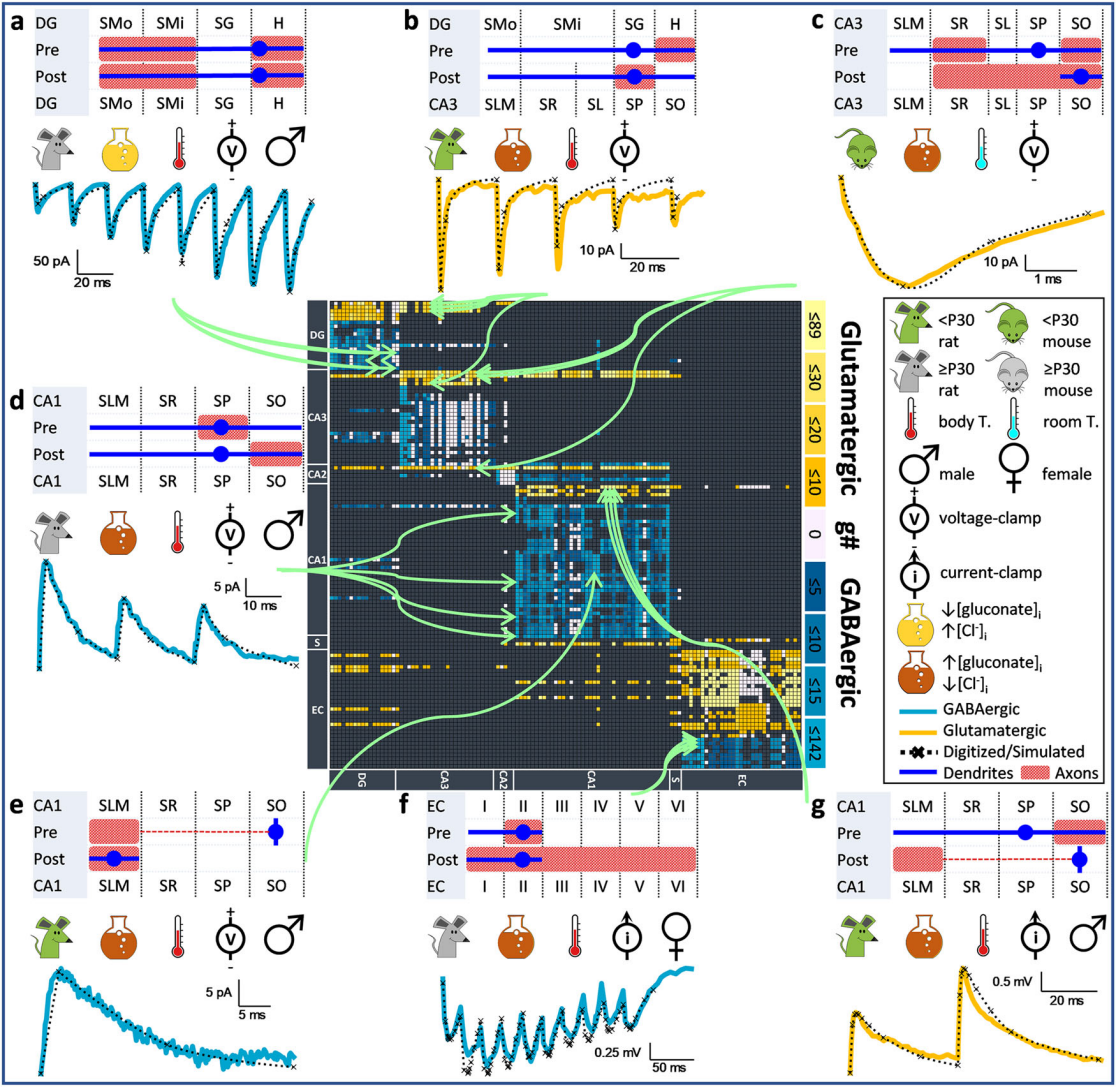
Diversity of synaptic covariates and mapping degeneracy. Examples of synaptic signals mined from peer-reviewed studies (solid lines: blue, GABAergic; gold, glutamatergic) and corresponding digitized reconstruction (dotted lines). Each signal is mapped to the possible presynaptic and postsynaptic neuron types (schematic morphologies; SL, lucidum). We only illustrated the most likely neuron types. The green arrows point to all possible mappings for every signal into the matrix of 3,120 potential connections (rows: presynaptic, columns: postsynaptic) among 122 neuron types. Blue and gold brightness in the connectivity matrix indicate the number of available experimental recordings. Light pink entries are potential connections with missing synaptic data. Black entries mark the absence of potential connection. The icons illustrate a sample of experimental covariates: species, age, sex, recording temperature and modality, and relative intracellular anionic concentrations. **a** Recording between a pair of dentate gyrus (DG) MOLAX or DG Total Moleculare Layer interneurons^75^. **b** signal from a DG Granule cell (or CA3 Granule, DG Semilunar Granule, or DG Hilar Ectopic Granule cell) to a CA3 Basket CCK+ cell^76^. **c** signal from a CA3 Pyramidal (or CA3c Pyramidal cell) to a CA3 Trilaminar (or CA3 Interneuron Specific Oriens) cell^77^. **d** signal from a CA1 Basket CCK+ (or CA1 Radial Trilaminar, CA1 Oriens/Alveus, or CA1 Schaffer Collateral-Associated) cell to a CA1 Pyramidal cell^78^. **e** signal from a CA1 O-LM cell to a CA1 Neurogliaform cell^79^. **f** signal from an entorhinal cortex (EC) LII Basket-Multipolar (or EC LII Axo-Axonic or medial EC LII Basket) cell to a medial EC LII Stellate neuron^80^. **g** signal from a CA1 Pyramidal cell to a CA1 O-LM (or CA1 Recurrent O-LM, or CA1 O-LMR) cell^81^.

We compiled, digitized, and reconstructed from the published literature a comprehensive dataset of 2,621 synaptic signals recorded from the dentate gyrus, CA3, CA2, CA1, subiculum, and entorhinal cortex^11^. For each recording, we annotated the detailed experimental conditions with 75 covariates (Methods; Table 1) and mapped the potential pair of presynaptic and postsynaptic neuron types among 3,120 potential connections identified by Hippocampome.org^9,10^. While this synaptic database constitutes a uniquely information-rich resource, its quantitative analysis requires solving distinct challenges (Fig. 2). First, researchers record synaptic signals in different modalities (current- or voltage-clamp) and widely diverse experimental conditions, which cannot be directly compared. Second, synaptic measurements can rarely be ascribed to single identified presynaptic and postsynaptic neuron types: in most cases, the mapping is ‘fuzzy’ and matches several potential connections (green arrows in Fig. 2). Third, synaptic data are unavailable for a sizeable minority of potential connections. Additionally, certain experiments only include one synaptic event (e.g., the upper right signal in Fig. 2), thus providing no information on short-term plasticity. We fit all synaptic recordings to the same model via signal simulation to solve part of the first challenge (normalizing recording modality and a subset of covariates). To solve the remaining challenges (normalizing the rest of the covariates, disambiguating potential connections, and inferring missing data), we devise an original strategy based on machine learning.

**Table 1:** List of features used for machine learning.

Features	Dimensions	Columns
Potential presynaptic neuron(s)	122 neuron types (one-hot encoded)	122
Potential postsynaptic neuron(s)	122 neuron types (one-hot encoded)	122
Stimulation method	Evoked, unitary, spontaneous, or miniature	3
Type of response	GABAergic or glutamatergic	1
Response contamination with another neurotransmitter	True vs false	1
GABA_B_ or NMDA response or block	Intracellular block, extracellular block, pure slow response, contaminated with AMPA or GABA_A_, and discoverable but was absent	5
Preservation of intracellular monoamines	True vs false	1
Calcium-permeable AMPA density	High, low, absent, or blocked	4
*E*_rev_	AMPA, NMDA, GABA_A_ and GABA_B_	4
*V*_m_	Values in mV	1
Temperature	Values in °C	1
Inter-stimulus interval	Values in ms, NA (0)	1
Species	Rats (1), mice (−1), or guinea pigs (0)	1
Sex	Male (1), female (−1), or unstated (0)	1
Postnatal age	Values in days	1
Slice region	Hippocampus, entorhinal cortex, dorsal, ventral, and [medial or lateral]	5
Slice orientation	Coronal, sagittal, transverse, horizontal, longitudinal, and ‘magic cut’	6
Slice thickness	Values in μm	1
Postsynaptic recording subregion	Soma, dendrite, or axon initial segment	1
Ionic concentrations of intracellular and extracellular solutions	Intracellular and extracellular values in molars corrected for the ionic activity constants of solutions (Ca, Mg, Na, K, Cl, Cs, Br, Ba, H_2_PO_4_, HPO_4_, HCO_3_, gluconate, QX314, ATP, GTP, EGTA, OH, SO_4_, phosphocreatine, acetate, methylsulfate, NMDG, tris, CeSO_4_, pyruvate, TEA)	36
Potency	True vs false	1

### Modeling comparable synaptic parameters from diverse measures and modalities

**Fig. 3:**
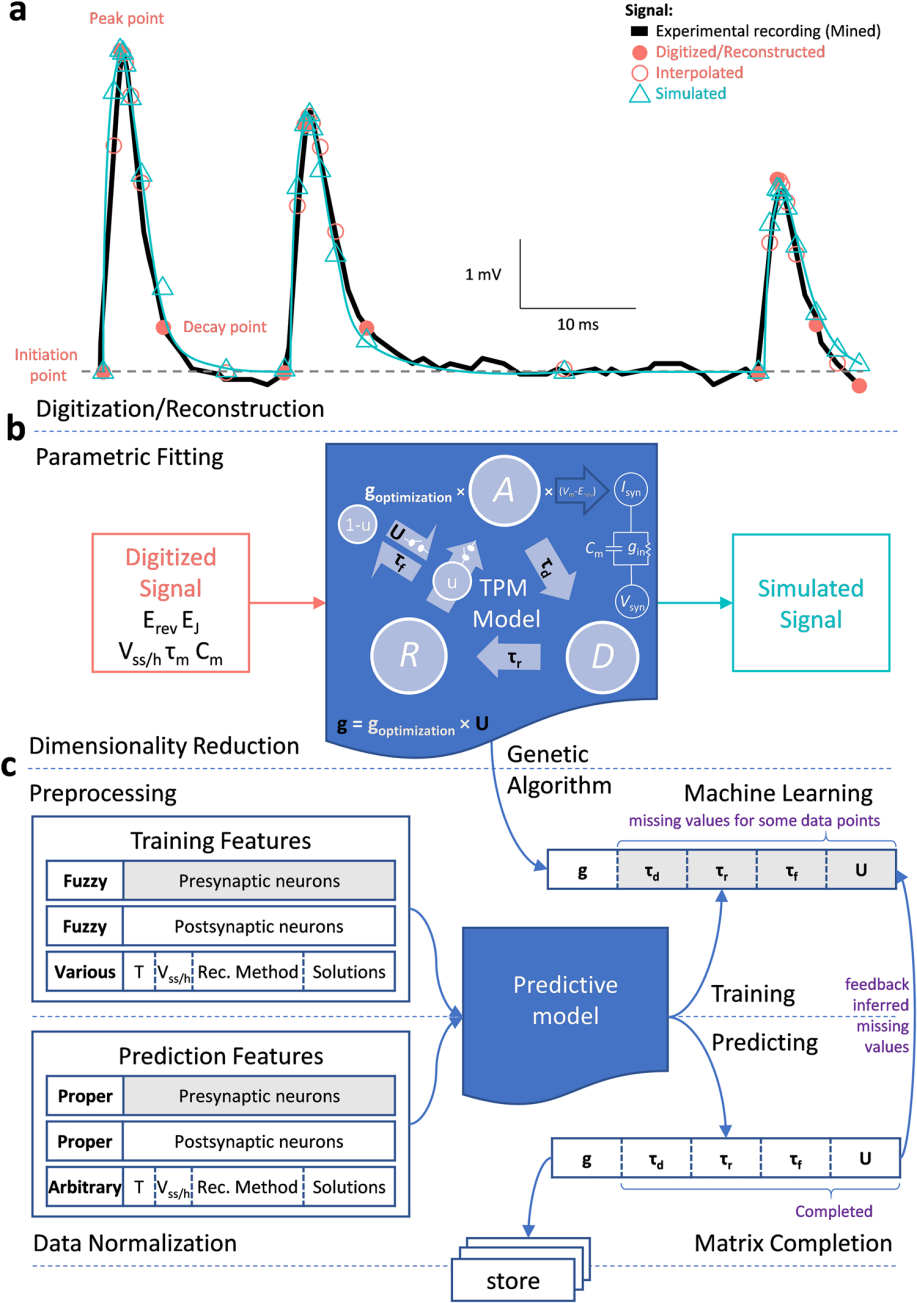
Synaptic modeling and deep learning. **a** Digitized trace (black line) from a CA2 Pyramidal cell to a CA2 Narrow-Arbor Basket PV+ cell^82^. The corresponding 9-point reconstruction of each spike (red circles), based on initiation, peak, decay (filled circles), and 6 interpolations (hollow circles), are used to optimize the simulated signal (green). **b** The Tsodyks-Pawelzik-Markram (TPM) model describes synaptic amplitude, kinetics, and short-term plasticity in terms of utilization rate (u), activation (A), deactivation (D), and recovery (R) dynamics. State A represents the portion of activated synapses; state D corresponds to deactivated synapses that are still bound to neurotransmitter and therefore cannot be reactivated; R is the portion of synapses detached from neurotransmitter and ready to be reactivated. The kinetics of the transition from A to D is determined by the synaptic decay constant *τ*_d_. The recovery rate is instead mostly determined by *τ*_r_. In terms of postsynaptic ionotropic neurotransmitter receptors, the TPM model assumes that ligand-gated channel opens instantaneously. The portion of recovered synapses that are instantly activated after a synaptic event is indicated by u (lower case). During deactivation, the gate is closing while the neurotransmitter is still attached to the channel receptor. For a full recovery, the neurotransmitter needs to detach from the receptor for presynaptic reuptake. The time constant *τ*_r_ measures that recovery speed. While *τ*_d_ mainly defines synaptic decay, it can affect ST-P as well when presynaptic firing is very fast. TPM model combined with Ohm’s law can simulate voltage-clamp experiments (synaptic current, *I*_syn_) by using experimentally measured reversal potential (*E*_rev_), junction potential (*E*_j_), and holding potential (*V*_h_). To simulate current-clamp experiments (synaptic potential, *V*_syn_), we fed the *I*_syn_ to a simple membrane model (Resistor-Capacitor circuit), which depends on experimentally measured steady-state potential (*V*_ss_), membrane capacitance (*C*_m_), and membrane time constant (*τ*_m_, that could be calculated knowing *C*_m_ and input conductance, *g*_in_). A genetic algorithm yields the best-fitting values of 5 synapse-specific parameters: conductance (*g*), single-exponential decay time constant (*τ*_d_), recovery time constant (*τ*_r_), facilitation time constant (*τ*_f_), and utilization ratio (*U*, capital letter). **c** The predictive machine learning model of synaptic electrophysiology used a deep learning architecture with five hidden layers and error backpropagation. The input layer encoded the (typically fuzzy) presynaptic and postsynaptic neuron types (122 × 2 nodes) and all covariates (75 nodes). The output layer consisted of one node for each of the 5 synapse-specific parameters. Model training used the best-fitting TPM parameters corresponding to the available 2,621 reconstructed traces and matching covariates (corresponding to ∼84% of 3120 potential connections). The model outputs the five predicted synapse-specific parameters for any directional pair of neuron types and desired choice of covariates. We used random forest to complete the missing ST-P values (*τ*_r_, *τ*_r_, and *U*) in training data, and deep learning to predict synaptic properties of potential connections for which no data existed, including experimental conditions and potential connections that have never been studied before.

Data integration starts with the digitization of published synaptic recordings (Fig. 3a). These signals are diverse in terms of measurement modalities (current vs voltage) and the composition of intracellular and extracellular solutions affecting reversal potentials (E_rev_). To transform these data into a comparable form, we fitted all digitized signals to a simplified Tsodyks, Pawelzik, and Markram (TPM) model, representing synaptic properties with five parameters (Methods)^12,13^. These synapse-specific parameters (g, *τ*_d_, *τ*_r_, *τ*_f_, and U) depend on the combination of presynaptic and postsynaptic neuronal types involved and are estimated by fitting the TPM model output to the digitized signals (Fig. 3b). The model also requires a small set of measurements that depend on experimental settings and the properties of the postsynaptic neuron: E_rev_, the initial value of the membrane voltage (V_m_), membrane time constant (*τ*_m_), and capacitance (C_m_). We corrected the signals before parametric fitting to eliminate the impact of processes causing slow signal fluctuations (Suppl. Figure 1a-b and Methods). The fitting quality was satisfactory resulting in minimal optimization error (Suppl. Figure 1c-left). However, we could find a weak correlation between paired-pulse ratio and the optimization error suggesting the TPM model simulates depressing synapses better than facilitating synapses (Suppl. Figure 1c-right). The TPM model produced comparable synaptic parameters and normalized the data with respect to synaptic driving force (V_m_ - E_rev_) by converting synaptic amplitudes to conductance. Overall, the process reduces data dimensionality by describing every signal with only five values.

### Construction and validation of a predictive model of all synapses

The fitted parameters for matching potential connections in different experimental conditions reveal a large degree of variation that could be associated with covariates such as animal sex, species, recording and stimulation methods, and temperature (Suppl. Fig. 2a–d). To normalize the effect of covariates, we trained a predictive deep learning model of the synaptic parameters using a five-layer autoencoder perceptron architecture (Fig. 3c and Suppl. Fig. 3; Methods section). Given a potential connection and experimental covariates (i.e., features: Table 1), the models learned to infer the five synaptic parameters (i.e., targets). Training converged to stable performance with learned values deviating on average less than 30% from the experimental measurements (Suppl. Fig. 4a). The model displayed no overfitting and the predicted values (for targets not included in the training set) deviated only marginally more (~32%) from the original measurements (Suppl. Fig. 4a).

**Fig. 4:**
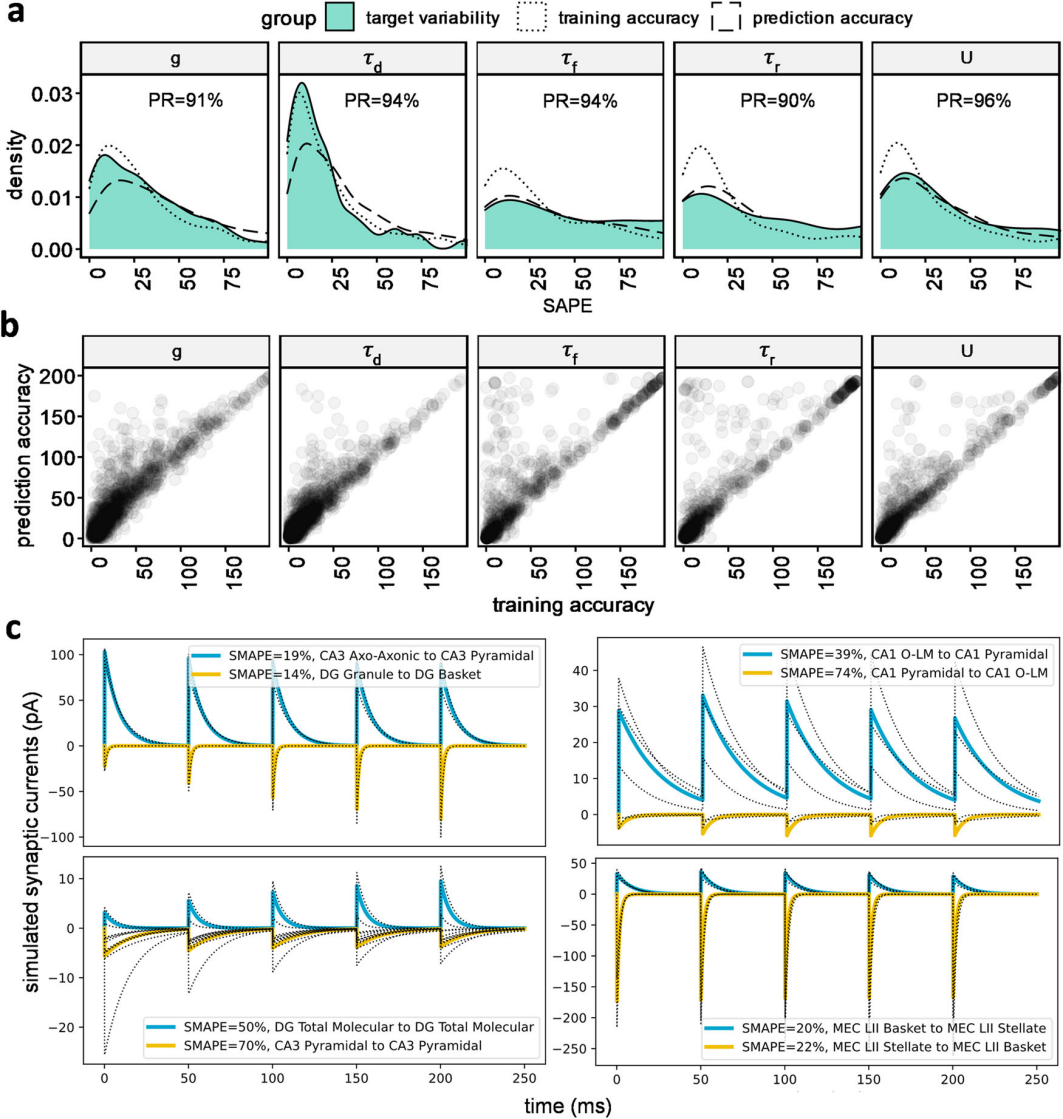
Validation and representative output of the deep learning model for unitary signals. **(a)** Probability distribution functions of the mismatch between model output and experimental data for each of the 5 TPM parameters (targets). The mismatch is quantified by the distance, measured by symmetric absolute percentage error (SAPE), of the model output relative to targets used during training (*training accuracy*) and targets left out of training set (*prediction accuracy*). These two measures to the intrinsic variability of the available experimental data grouped based on features (*target variability*). Synapses are plastic and stochastic entities, hence if neuron pairs from the same presynaptic and postsynaptic types are recorded several times in the same conditions, several values will typically be obtained experimentally, while model outputs will be constant. In other words, target variability is the distance of a training data point from the average of targets with identical features. This measure of variability in the experimental dataset defines the ideal limit of model accuracy. Training accuracy quantified the learning capacity of the model. Prediction accuracy measures the inference power of the model by calculating the distance of model output with a ground truth, which is the experimental data not seen by the model during jackknife (leave-one-out) procedure. The overall similarity of distributions indicates that the model achieved a level of accuracy comparable to the reproducibility of corresponding experimental data. The prediction reliability (PR) is the proportion of model outputs falling within the 95% confidence interval of the experimental data with identical features. **(b)** Prediction and training accuracy are highly correlated for all 5 parameters, suggesting the absence of overfitting. **(c)** Simulated synaptic dynamics based on original training targets (black dotted traces) with identical features, and deep learning model inferences (solid lines). Model predictions are remarkably close to the training data even though experimental data showed variability. Simulated synaptic traces (V_h_ = −35 mV, GABA_A_ E_rev_ = −70 mV, and AMPA E_rev_ = 0 mV) showed a wide range of amplitudes and kinetics as well as different forms of short-term plasticity. Glutamatergic and GABAergic examples are provided for every area involved in the tri-synaptic circuit.

To assess model performance, we calculated training and prediction accuracies for the five synaptic parameters over all the data. Training accuracy measures how well the deep learning model fits the synaptic parameters for a given pair of neuron types and a specific set of experimental covariates that were included in the training data. Prediction accuracy measures how well the deep learning model predicts the synaptic parameters for the data that were excluded from the training set. To assess this performance relative to the reliability of experimental measurements, we consider different experimental values (targets) recorded from the same nominal conditions (features). Those differences can be ascribed to unknown experimental factors, intrinsic biological variability, and random noise. We take such empirical ground-truth range as the gold standard to benchmark our model against. In these cases, we calculated the distance of each target value from their average, a measure of experimental fluctuation we call target variability (Fig. 4a and Suppl. Fig. 4b). The within-group sample size, summarizing the number of identical features with multiple measurements (*n* values in Suppl. Fig. 4d) ranged from 2 to 10.

We compared the target variability with the training accuracy and prediction accuracy, i.e., the distance of model output from seen and unseen targets, respectively. The training and prediction accuracies of our predictive model were remarkably close to the target variability. Testing the predictive power of the model with the jackknife (leave-one-out) method, we found that the vast majority of unitary predictions fell within the 95% confidence interval of the targets, i.e., they were reliable (Fig. 4a, b). Specifically, this prediction reliability ranged from 90% for *τ*_r_ to 96% for *U*, with intermediate values for *g* (91%), *τ*_d_ (94%), and *τ*_f_ (94%). By including all synaptic measurements (not just the unitary values, prediction reliability was reduced slightly to 88–94% (Suppl. Fig. 4b–d). Additionally, comparing the relevant values to sparse estimates available for matching potential connections from a recent CA1 study^14^ revealed no statistically significant difference for any of the five parameters (Suppl. Fig. 5a). The paired-pulse ratio of the models and the data before and after data normalization were also correlated, indicating both the TPM and the deep learning models had a reliable fit to the data (Suppl. Fig. 5b). Thus, the deep learning model quantitatively predicts the properties of synaptic signals for which experimental recordings are available within the margin of measurement accuracy.

### Connectivity matrix completion and synaptic data normalization

Given its demonstrated performance on available data, the predictive model can confidently estimate the synaptic parameters of yet uncharacterized potential connections based on the learned properties of neuronal types. The model can complete the synaptic electrophysiology matrix for all 3120 potential connections in the hippocampus and entorhinal cortex. Additionally, since the learned neuronal properties are all unique, the model also effectively disambiguates each potential connection: in other words, the predicted synaptic parameters for each pair of neuron types are also all unique. Notably, the deep learning model can infer synaptic parameters for every potential connection in any desired condition. Applying homogeneous conditions for all potential connections practically normalizes the inferences with respect to the covariates. This study primarily focuses on fast unitary synaptic properties in near-physiological (henceforth standard) conditions, namely AMPA and GABA_A_ synapses of adult male rats in voltage-clamp at body temperature and with a pipette solution that does not disturb intracellular ionic concentrations (Methods). These so-derived synaptic signals reflected the training data and showed a wide range of amplitudes, kinetics, and ST-P across potential connections (Fig. 4c and Suppl. Movie 1). To explore regional differences within the hippocampal formation, we inspected the probability density distributions of all parameter values normalized using the min-max method (Suppl. Fig. 6a). Interestingly, the range of values in the entorhinal cortex is smaller than in the hippocampus. Moreover, the GABAergic and the glutamatergic synapses had overlapping distributions for g and U but not for the time constants (Suppl. Fig. 6b), suggesting that these synapse types have similar amplitudes but differ in decay kinetics and ST-P.

### Open access to data and source codes

**Fig. 5:**
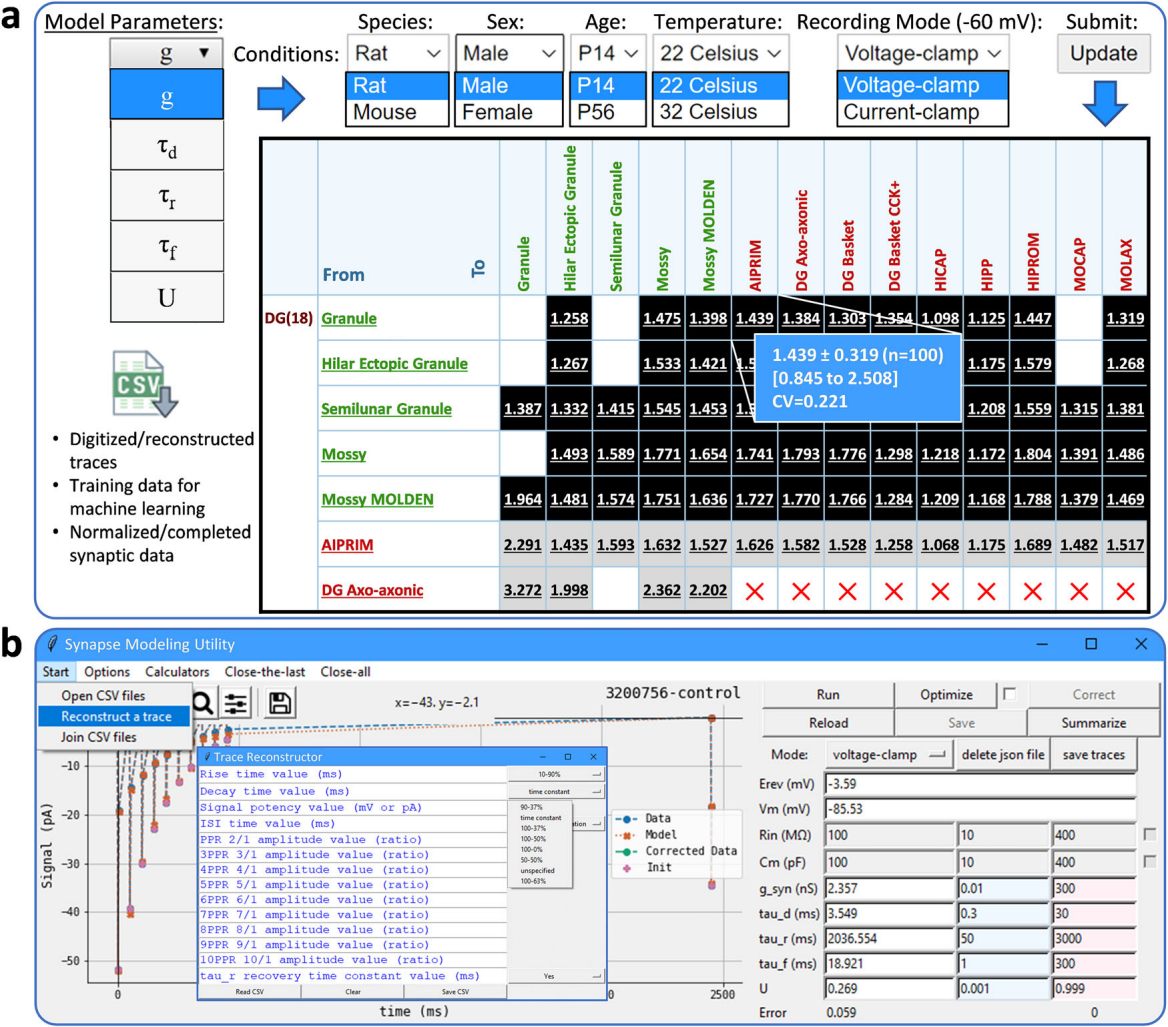
Comprehensive open access to data and tools. **a** The full set of normalized synaptic data for the entire entorhinal-hippocampal circuit in 32 different conditions (any combination of rat/mouse, male/female, young/adult, body/room temperature, and voltage-/current-clamp), the reconstructed synaptic traces with original references and annotated metadata, and the machine learning training data are all freely available at Hippocampome.org/synapse. For each synaptic parameter of every potential connection, we supply the mean, standard deviation, and range of 100 deep-learning model predictions. **b** Our high-performance synapse modeling tool (Hippocampome.org/SynapseModelingTools) is equipped with a Trace Reconstructor and trace correction algorithm.

The normalized and completed synaptic data are broadly applicable to designing experiments in optimal conditions, testing hypotheses, constraining biologically plausible simulations of the entire entorhinal-hippocampal circuit^23^, and benchmarking machine learning algorithms. We provide five synaptic constants for each of 3120 connections in 32 different settings that include all binary combinations of species (rat or mouse), sex (male or female), age (young or adult), recording method (voltage- or current-clamp), and temperature (room or body). For each parameter we make available the mean, standard deviation, and range over 100 training runs of the deep learning model (Fig. 5a). We also share all implemented tools for unhindered reuse with other datasets. The Synapse Modeling Utility, the preprocessing and analysis code in R, the machine learning library in Python, and the preprocessed machine learning-ready experimental data (2621 features-targets sets) are all freely available on Hippocampome.org/synapse (Fig. 5b).

### Presynaptic and postsynaptic determinants of synaptic physiology

**Fig. 6:**
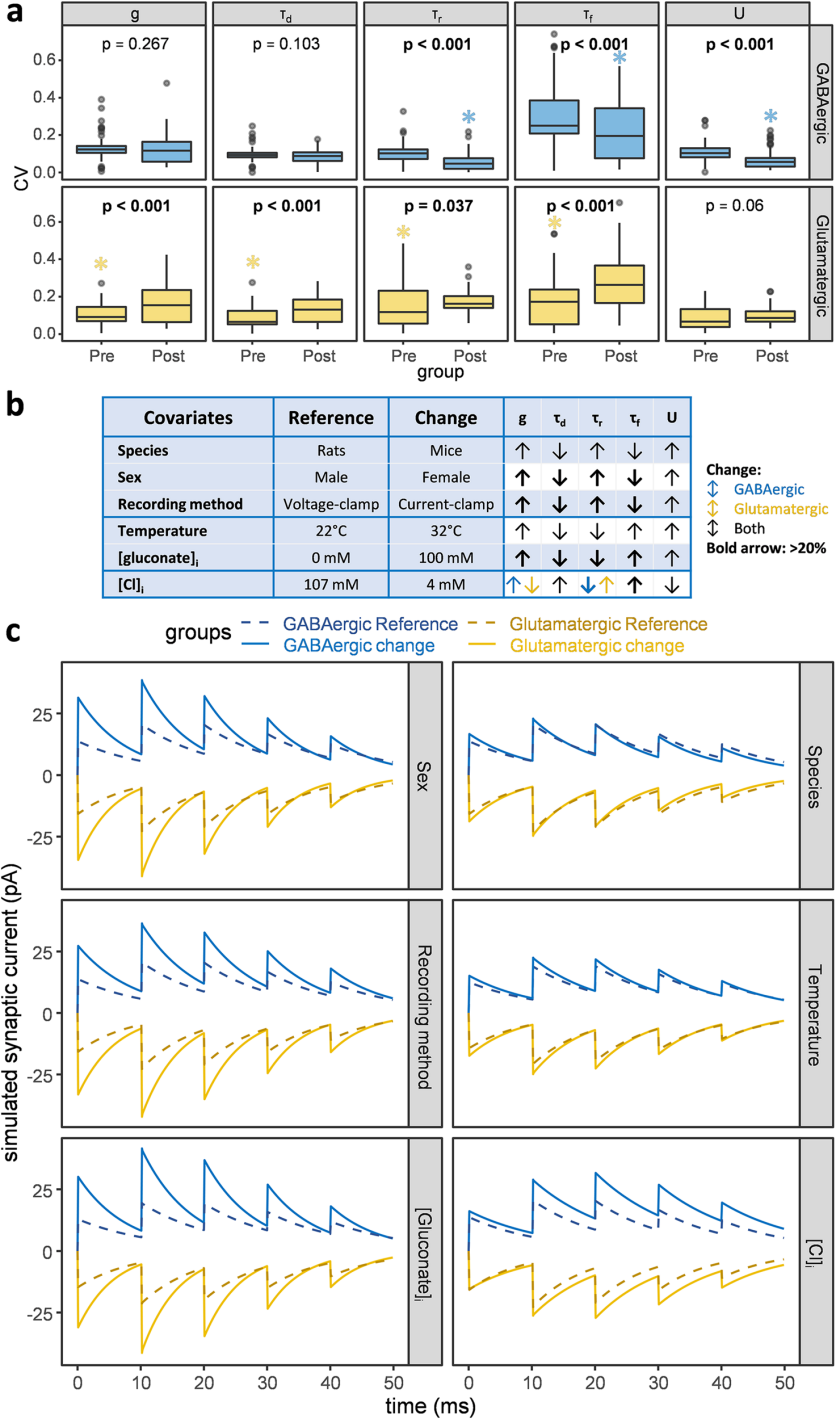
Principal determinants of synaptic properties. **a** To ascertain the relative importance of presynaptic axons and postsynaptic dendrites on synaptic dynamics, we measured the coefficient of variation (CV) of all 5 parameters for each (presynaptic or postsynaptic) neuron type across its potential connections. We then assessed the difference between these two groups by unpaired Wilcoxon test. A significantly lower variability (asterisks) indicates a dominant role of that group in defining the synaptic signal. The lower and upper hinges of the box and whiskers plot correspond to the first and third quartiles around median. The dataset underlying this analysis is available in Supplementary Data 1. **b** To investigate the impact of covariates on synaptic parameters, we changed one experimental condition at a time and assessed the differences by paired Wilcoxon test. All changes were statistically significant; bold arrows indicate differences >20%. **c** To simulate a consensus signal for each group in every pair of conditions, we averaged the 5 synaptic parameters across all relevant (GABAergic or glutamatergic) connections. Comparatively, sex, recording method, and [gluconate]_i_ had the greatest phenomenological impact (in these simulations, *V*_h_ = −30 mV, GABA_A_ *E*_rev_ = −60 mV, and AMPA *E*_rev_ = 0 mV).

Deep learning-enabled full data normalization allowed us to compare the synaptic properties of all potential connections without the influence of confounding variables. Hereunder, we analyzed the normalized data. We reported trends in unnormalized data in an earlier paper^11^. To begin investigating how the presynaptic and postsynaptic identities combine to define synaptic dynamics, we asked two questions: (1) when a pair of neuron types forms a synapse, which synaptic properties (e.g., amplitude, duration, ST-P) does either side dominantly determine? (2) Does the answer differ for glutamatergic and GABAergic synapses? To answer these questions, we separated the glutamatergic and GABAergic synapses. In each pool, we created two groupings: one based on the presynaptic neuron types, and the other based on the postsynaptic ones. For example, the glutamatergic presynaptic grouping consisted of 38 groups, one for every glutamatergic presynaptic type; each group contains all postsynaptic neuron types that presynaptic type forms a connection with. We then calculated for each group the coefficient of variation (CV) of all five synaptic parameters in the standard condition (Fig. 6a). A lower CV indicates less intragroup variation and thus a tighter control of the corresponding grouping on that synaptic property. For GABAergic synapses, the ST-P parameters (*τ*_r_, *τ*_f_, and *U*) had significantly smaller CVs if synapses were grouped based on postsynaptic type. In contrast, for glutamatergic synapses, all parameters except U had significantly smaller CVs if synapses were grouped based on presynaptic type. In other words, presynaptic glutamatergic neurons and postsynaptic GABA_A_ receptors are more important determinants of synaptic signals.

### Principal covariate effects on synaptic properties

Next, we systematically investigated the influence of experimental covariates on synaptic parameters. Earlier research mainly checked the impact of experimental conditions on synaptic amplitude and kinetics of a limited number of neuron types. Our study also allowed the inclusion of ST-P parameters and systematically covered all potential connections of the hippocampal formation by changing one covariate at a time. All tested covariates had a statistically significant impact on synaptic parameters, but we only report here (Fig. 6b, c) those with a meaningful effect size (>10%) and emphasize the most substantive ones (>20%). Our results indicate that *g* increases more than two-fold and *τ*_d_ decreases 30% when switching from voltage- to current-clamp, from male to female animals, and from gluconate-free to gluconate-containing intracellular solutions. While the change with recording modality agrees with previous studies, for example^24^, and we expected a difference by sex, the pronounced impact of gluconate in the pipette solution was surprising. Moreover, current-clamp (relative to voltage-clamp) and female animals (relative to male) also entailed notably higher *τ*_r_ and lower *τ*_f_, implying greater propensity towards synaptic depression. In contrast, the opposite trend, conducive to facilitation, was observed with gluconate. Shifting from rats to mice or from room to body temperature affected synaptic properties in the same direction, but to a more modest extent (10–20% effect size), as the male-to-female switch or intracellular gluconate addition, respectively. Reducing [Cl]_i_ substantially increased short-term facilitation at GABAergic synapses, while more modestly slowing down synaptic kinetics which was unexpected based on^25^. Other covariates, including to our surprise age, did not affect the parameters substantially. Altogether, remarkably, only two types of variation, differing just in the change direction of *τ*_r_ and *τ*_f_, could explain the impact of all analyzed covariates irrespective of neurotransmitter type. This observation suggests an interdependence among synaptic parameters.

### Synaptic amplitude predicts signal kinetics and the direction of short-term plasticity

**Fig. 7:**
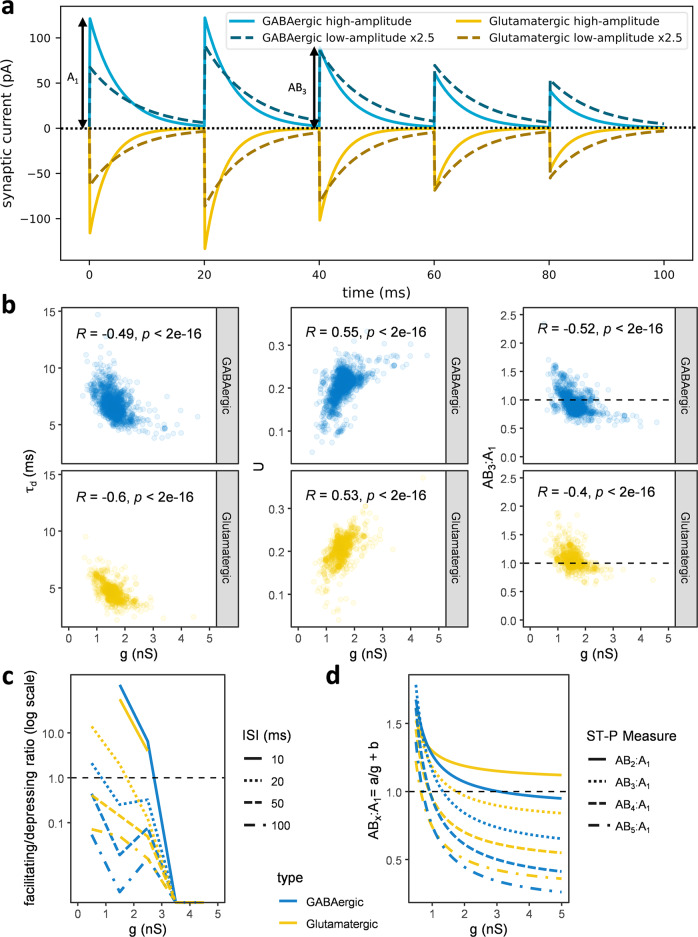
Synaptic conductance entails kinetics and short-term plasticity. **a** We averaged the synaptic parameters of the 30 potential connections with the largest conductance (high-amplitude) and of the 30 with the smallest one (low-amplitude) to compare their simulated consensus signals. The low- and high-amplitude groups exhibited respectively short-term facilitation and depression (ISI = 20 ms, *V*_h_ = −35 mV, GABA_A_ *E*_rev_ = −70 mV, and AMPA *E*_rev_ = 0 mV). A_1_ is the synaptic amplitude of the first event; AB_3_ is the amplitude from the baseline of the third event. **b** Considering all connections, synaptic amplitude correlates with kinetics and short-term plasticity (ST-P). The negative correlation between *g* and *τ*_d_ (left) indicates that synapses with large conductance tend to have faster signal decays. The positive correlation between g and U (middle) suggests that high-amplitude synapses have higher resource utilization. The negative correlation between *g* and the ratio from the baseline of the third event, AB_3_:A_1_, (right) shows that high-amplitude synapses depress more. **c** The ratio between facilitation (AB_3_:A_1_ > 1) and depression (AB_3_:A_1_ ≤ 1) decreases as a function of conductance with a transition from mainly facilitating to mainly depressing between 1 nS (GABAergic) and 1.7 nS (glutamatergic) for 20 ms ISI. **d** Fitting the dependence on *g* of the ratio from the baseline of the x^th^ event, AB_x_:A1, with an inverse first-order polynomial function reveals that earlier synaptic events (e.g., AB_2_:A_1_) tend to facilitate while later ones (e.g., AB_5_:A_1_) tend to depress. The trend from facilitation to depression with increasing conductance is robust to ST-P measure.

Among both glutamatergic and GABAergic types, we noticed that synapses with high amplitude had fast kinetics and demonstrated depressing ST-P. Conversely, synapses with low amplitude had slower kinetics and were facilitating. To visualize these observations, we averaged the model parameters from the 30 synapses with the largest conductance and from the 30 with the smallest one among both glutamatergic and GABAergic groups. We then compared the responses of the four consensus models in standard conditions (Fig. 7a and Suppl. Movie 1). The high-amplitude models exhibited short-term depression and short signal duration (half-height width: 2.4 ms for glutamatergic and 3.8 ms for GABAergic), while the low-amplitude models demonstrated short-term facilitation and long signal duration (half-height width: 5.1 ms for glutamatergic and 6.2 ms for GABAergic). Considering all 3120 connections revealed a significant negative correlation between *g* and *τ*_d_ and between g and the paired-pulse ratio from baseline of the third synaptic event (AB_3_:A_1_), but a positive correlation between *g* and *U*, suggesting that high-amplitude synapses have higher resource utilization (Fig. 7b). Facilitation and depression partly depend on interstimulus intervals (ISI) and the measure of ST-P. Testing ST-P at 20 ms ISI and considering AB_3_:A_1_, the majority (>90%) of synapses with an amplitude below 0.5 nS facilitated, irrespective of neurotransmitter, while most synapses above 2 nS (glutamatergic) or 3 nS (GABAergic) depressed (Fig. 7c, left). Although the second synaptic events (AB_2_:A_1_) tended towards facilitation relative to subsequent signals (e.g., AB_5_:A_1_), all ST-P measures consistently transitioned from facilitation to depression as a function of conductance (Fig. 7c, right). Moreover, *τ*_f_ and *τ*_r_ were negatively correlated (*R*_glu_ = −0.4, *R*_GABA_ = −0.1, *p* < 0.05), indicating that synapses needing a long time to recover their resources tend to reduce their synaptic utilization rate rapidly. Altogether, these analyses suggest that higher synaptic amplitudes predict faster kinetics and a tendency towards depression over facilitation, reflecting coordinated differences in *τ*_d_ and *U* as well as interdependence of *τ*_f_ and *τ*_r_.

### Presynaptic and postsynaptic molecular expression as a biomarker of short-term plasticity

**Fig. 8:**
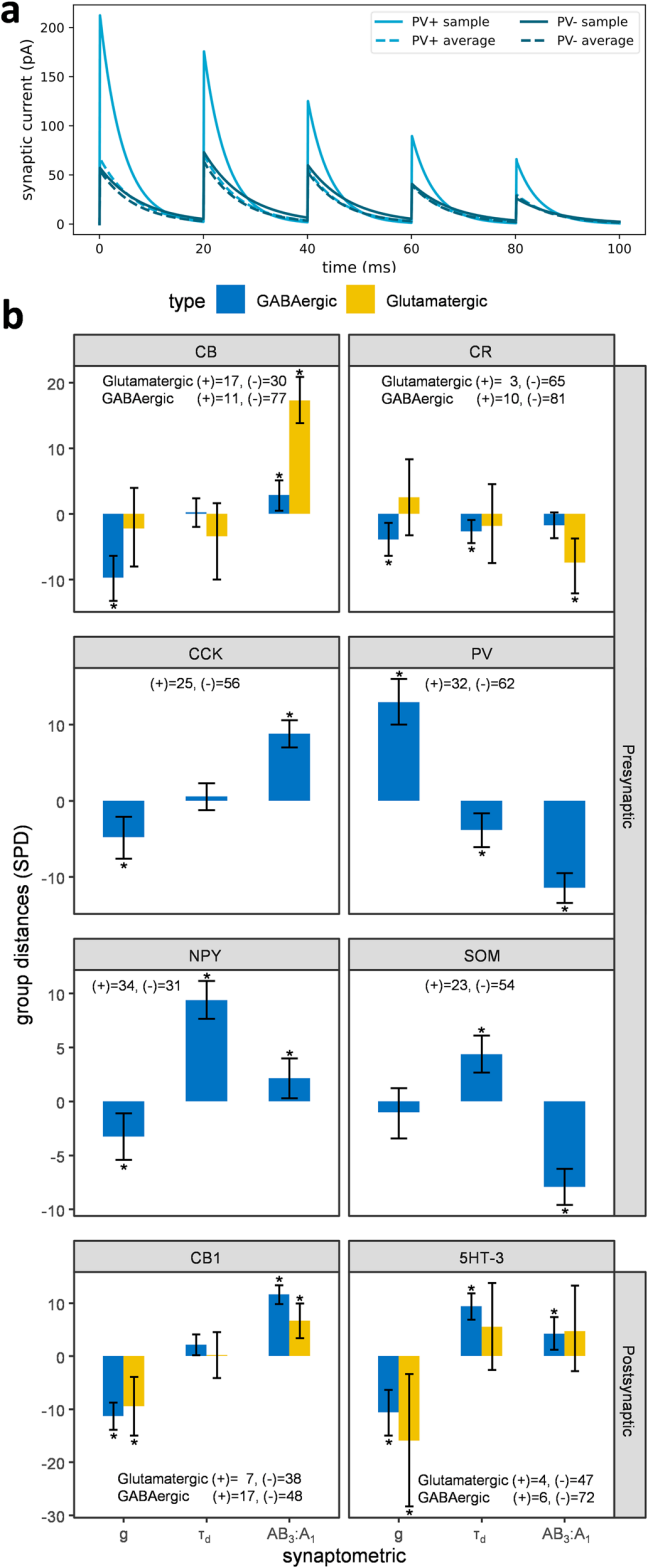
Presynaptic and postsynaptic molecular markers of synaptic physiology. **a** Comparison of synaptic signals between presynaptic neuron types expressing (+) or not expressing (−) parvalbumin (PV). The consensus trace was simulated with synaptic parameters averaged across all potential connections in each group. The sample traces are from the CA1 Basket PV+ to CA1 Pyramidal connection (+), and from CA1 Basket CCK+ to CA1 Pyramidal (−). **b** Symmetric percentage differences (SPD) in synaptic parameters between potential connections grouped by the expression or absence of specific presynaptic or postsynaptic molecular markers (with corresponding samples sizes). Positive values signify that the measurement is larger in the (+) than in the (−) group, and vice versa. Error bars indicate confidence intervals and asterisks denote statistical significance by unpaired Wilcoxon test at the *p* < 0.05 level after False Discovery Rate correction^63^ for multiple testing. The dataset underlying this analysis and the listing of all 36 p-values are available in Supplementary Data 1.

It is a widespread practice to study synapses based on molecular expression. Chemical biomarkers were not directly among the training features of our predictive synapse models, but were used for mapping mined signals to potential connections^11^. Since the normalized inferences are not fuzzy, we employed Hippocampome.org to query neuron types expressing different markers^26,27^ and analyzed differences in synaptic properties among neuron types containing (+) or lacking (−) each molecule. Since marker expression can be localized to the presynaptic terminals or postsynaptic dendrites and soma^28^, we studied the presynaptic and the postsynaptic groups separately (Fig. 8). Considering AB_3_:A_1_ as a measure of ST-P and using a 20 ms ISI, we identified two classes of presynaptic markers that respectively predicted synaptic facilitation and depression. Specifically, presynaptic calbindin (CB), cholecystokinin (CCK), and neuropeptide-Y (NPY) expression correlated with facilitation (larger AB_3_:A_1_ values).

In contrast, calretinin (CR), parvalbumin (PV), and somatostatin (SOM) correlated with depression (smaller AB_3_:A_1_ values). The relations of these markers with changes in synaptic amplitude and kinetics were not always statistically significant but generally followed the trends revealed in the previous section. Namely, presynaptic expressions predicting short-term facilitation typically demonstrated lower signal amplitudes and slower kinetics and vice versa for those predicting short-term depression. Cannabinoid receptor 1 (CB1) expression can be localized both on presynaptic and postsynaptic sides of a synapse^29^. Since the presynaptic effects were similar to CCK, we only illustrated the postsynaptic effects. Among the postsynaptic markers, CB1 and serotonin receptor 3 (5HT-3) predicted lower amplitudes and a tendency towards facilitation. Interestingly, CB1 changed g and AB_3_:A_1_ of GABAergic synapses more than of glutamatergic synapses, on average.

### Correlations between neuronal morphology and synaptic parameters

In GABAergic neurons of both hippocampal area CA1 and visual cortex, the kinetics of spontaneous synaptic inputs vary depending on the specific axonal targeting of that same postsynaptic neuron^30,31^. We tested similar interactions between input synaptic properties and output axonal patterns throughout the hippocampal formation, not only considering unitary synaptic kinetics, but also conductance and ST-P (Suppl. Figure 7). Among GABAergic synapses in CA1, we found significant differences in *g*, *τ*_d_, *τ*_f_, and *U*, indicating that input synaptic duration, as well as amplitude and facilitation, vary by output axonal targeting (Fig. S7a). Extending the study to other hippocampal regions revealed significant differences in *τ*_d_ and *τ*_f_ among GABAergic synapses in CA3, and in *τ*_r_ in DG and CA2. Glutamatergic synapses generally demonstrated fewer significant differences. Visualizing consensus traces (Suppl. Figure 7b) and synaptometrics differences (Suppl. Fig. 7c) confirmed these patterns.

In the visual cortex, connection probability correlates with synaptic strength^32^. Hippocampome.org calculates the probabilities of connections and the average synaptic distance from the presynaptic and postsynaptic soma based on the layer-specific linear densities of the corresponding axons and dendrites^33^. Synaptic conductance had a weak but statistically significant positive correlation with the connection probability (*R*_GABA_ = 0.27, *R*_Glu_ = 0.19, *p* < 0.05). Consistent with dendritic filtering, we found a significantly negative correlation between g and the synaptic distance from the postsynaptic soma (*R*_GABA_ = −0.13, *R*_Glu_ = −0.06, *p* < 0.05).

## Discussion

We digitized, reconstructed, and compiled a comprehensive dataset of 2621 synaptic signals recorded from the rodent hippocampus and entorhinal cortex, and mapped each to respective covariates and potential connections. Through computational modeling and machine learning, we normalized and completed all synaptic physiology data to predict the amplitude, kinetics, and ST-P of the 3120 potential connections of the hippocampal formation. For each potential connection, we freely released via Hippocampome.org the complete set of 5 synaptic parameters in 32 different experimental settings with all annotated experimental data, plus analysis and modeling software source code. We identified the major determinants of unitary synaptic physiology and discovered new correlations among synaptic properties, molecular expression, and neuronal morphology.

Broad diversity in experimental settings causes extreme variability in synaptic electrophysiological recording. Combined with inherent measurement noise, this makes identifying causal relations among variables considerably challenging. To our knowledge, our application of deep learning provides a suitable solution to these issues. Testing the deep learning model with unseen data demonstrated that the predictions are valid within experimental accuracy. Applying uniform experimental conditions (voltage-clamp at body temperature in male rats with specified intra- and extra-cellular solutions) to all potential connections effectively normalized data. In that scenario, the only differences in synaptic parameters are due to the presynaptic and postsynaptic neuron identities. At the same time, changing the chosen experimental condition, such as switching from male to female animals, allows the systematic investigation of every covariate effect.

Furthermore, our deep learning solution yields two notable data augmentation benefits. First, it fills in missing data by matrix completion harnessing the learned axonal and dendritic properties of the corresponding neuron types. In simple terms, if the predictive model learns synaptic features from neuron type x to neuron type y, and from type w to type z, it can then infer the features from x to z and from w to y based on the axonal properties of x and w and the dendritic properties of y and z. In reality, the known features utilized in training are more numerous than the set of missing data. For comparison, an earlier study measured the synaptic physiology of 10% of potential connections in CA1 to extrapolate the properties of the remaining 90% based on marker profiles^14^. In contrast, our experimental dataset covered most potential connections across the entire hippocampal formation, with missing values ranging from 16.3% for conductance to 38.5% for ST-P. Singular value decomposition (SVD) may robustly complete matrices with up to 50% of missing values^34^, but deep learning typically outperforms SVD in this process^18^.

The second beneficial effect of our machine learning approach is that it leverages data redundancy to disambiguate the mappings of individual signals to multiple potential connections. Consider for instance an experimental recording mapped to potential connections A or B and a different recording mapped to potential connections B or C; the deep learning model utilizes the two constraints on B to predict a unique set of synaptic parameter values distinct from those of A and C. Indeed, the inferred values were all different for the 3120 pairs of hippocampal neuron types, indicating that the training data was sufficient to completely resolve degenerate mappings.

The method we introduced is highly flexible and can be adapted to include pathological conditions. For example, the set of features could be expanded by including a descriptor to distinguish the epileptic state. Then, mining and modeling available epileptic data, would allow the inference of normalized and completed synaptic properties in epilepsy. At this time, we have mined only the control condition of experiments. However, our freely accessible code and data allow any interested researcher to add and normalize their recordings as needed. The more data are pooled together, the more generalizable the resulting predictions will be.

Before delving into the implications of the results, we should note that our observations are circumscribed to the somatic impact of synaptic events. To investigate local postsynaptic mechanism likely requires compartmental modeling of dendritic morphologies, or optoelectrophysiological techniques^14,35^. We also did not consider long-term plasticity and slow conductances (GABA_B_ or NMDA receptors; but see Suppl. Note 1) since the required experimental data for most pairs of neuron types remain sparse. Our regression models likewise excluded stimulation strength of evoked events because this detail is seldom reported in publications. Additionally, most published traces had a constant ISI and a single recovery event. Future experimental designs including variable ISIs and multiple recovery events would allow more accurate estimation of *τ*_r_ and *τ*_f_. Since homeostatic plasticity may change synaptic strength in vitro, we suggest reporting systematically the time elapsed from slice preparation until the actual recording. It is also important to acknowledge that the chosen TPM formalism is a fairly simple analytical model. While it performs satisfactorily for the majority of synapse types in the hippocampal formation, more complex models accounting for rise time constant, calcium concentration, and stochasticity may be better suited for extremely facilitating synapses^14,36^ (see Suppl. Note 2).

The synapses of the entorhinal-hippocampal network communicate through a broad continuum of signal amplitudes. Yet, the sets of neurotransmitters and receptors employed by this circuit are limited, raising a question: does variation in synaptic conductance interact with resource utilization and recovery to affect kinetics and ST-P? Unnormalized unitary data suggest that decay kinetics are faster for strong GABAergic synapses than for weak ones^11^. Additionally, one study on three synapse types suggests that the ST-P of stronger synapses is depressing, and the ST-P of weaker synapses is facilitating^37^. Indeed, analyzing all potential connections of the hippocampal formation revealed a negative correlation of g with both *τ*_d_ and AB_3_:A_1_. Moreover, we found a positive correlation between g and U, consistent with the TPM model (Eq. 18 in Methods). Since U quantifies the utilization increment, these results suggest high-amplitude synapses depress more easily because of resource exhaustion.

The TPM model accounts for resource utilization and recovery. When *τ*_r_ is small, the resource recovery pace is fast. When *τ*_f_ is large, resource utilization remains prolonged. Therefore, the opposite dependence of *τ*_f_ and *τ*_r_ on covariates indicates that when resource recovery pace is fast, resource spending is prolonged. Furthermore, their higher negative correlation in glutamatergic synapses relative to GABAergic ones suggests that resource utilization is subject to tighter control in the former than in the latter. Overall, the effects of covariates on synaptic parameters revealed only two distinct patterns that differed exclusively in the change direction of *τ*_r_ and *τ*_f_. The correlation among synaptic parameters could explain the mere simplicity of these observations. Covariates increasing g will also increase *U* and decrease *τ*_d_. The only remaining freedom is in *τ*_r_ and *τ*_f_, which always change in opposite directions. This suggests that covariates affect a small set of latent variables. See Suppl. Note 3 for further discussions.

For equivalent experimental conditions and irrespective of the neurotransmitter, female animals had, relative to males, multiplicatively larger unitary synaptic conductance, significantly faster kinetics, and greater tendency towards short-term depression than facilitation. It is tempting to speculate a link to chronic exposure to neurosteroids and endocannabinoids, which increase the amplitudes of glutamatergic and GABAergic synapses, respectively, in females^38–40^. We observed similar changes in synaptic parameters when switching from voltage-clamp to current-clamp. This could be due to the activation of voltage-gated ion channels in current-clamp or the reduction of passive filtering during parametric fitting that brings the estimations closer to the local dendritic event^24,41^. We also found qualitatively parallel differences between species, with significantly larger synaptic conductance in mice compared to rats. Notwithstanding the high statistical sensitivity of our study, however, the phenomenological disparity across rodents was practically negligible (see Suppl. Note 4 for further considerations).

When added to the patch-clamp intracellular solution, the common food additive potassium gluconate (E577) changes the reversal potential of GABA_A_ channels^42^, blocks ion channels involved in subthreshold membrane physiology^43^, and alters firing patterns in hippocampal neurons^44^. However, the impact of intracellular gluconate on unitary synaptic signaling has never been studied systematically. We found intracellular gluconate to be one of the most potent synaptic enhancers. With gluconate in the recording pipette, synaptic amplitudes were a fold-factor larger, kinetics were faster, and short-term plasticity shifted from depression to facilitation (smaller *τ*_r_ and larger *τ*_f_). The increment of synaptic amplitude could be explained by blockage of the subthreshold channels, which reduces shunting and increases input resistance. The reduction of short-term depression may be due to the role of gluconate as an energy source that facilitates resource recovery. As a comparison, the effect of gluconate on synaptic parameters was a full order of magnitude larger than the changes observed in the same direction when shifting from room temperature to body temperature.

Our data analysis suggests that the presynaptic side of glutamatergic, and the postsynaptic side of GABAergic neurons, have a relatively higher impact on synaptic properties. For GABAergic synapses, this finding could be explained by the selective targeting of Axo-axonic and Interneuron-Specific interneurons^9^. At the same time, each neuron type in Hippocampome.org is linked to known molecular biomarkers expressed either in the axons (e.g., calcium-binding proteins and neuropeptides) or in the dendrites (e.g., neurotransmitter receptors). Among calcium-binding proteins, calbindin was a biomarker of facilitating synapses while calretinin and parvalbumin of depressing ones. Among neuropeptides, CCK and NPY marked a tendency toward facilitation and somatostatin towards depression. Among neurotransmitter receptors, cannabinoid receptor 1 and serotonin-gated ionotropic channels altered synaptic properties similarly. While this result is consistent with their pattern of co-expression in cortical neurons^45^, their underlying mechanisms are likely distinct given the specific dendritic compartmentalization of 5-HT3, but not of CB1. See Suppl. Note 5 for further discussions.

Normalized synaptic data are required by large-scale modeling efforts, such as the European Union Human Brain Project. Using our approach, experimental synaptic recordings can be properly integrated by computational modeling and deep learning to provide the much needed normalized, completed, and disambiguated unitary electrophysiology data of all potential connections in the hippocampal formation in any desired setting. These data can be used to test hypotheses, constrain and validate realistic computer simulations, and optimize experimental designs. The hippocampal formation is a current focus of broad community interest, but our platform can be applied to other brain regions and circuits as well. The devised method and tools can facilitate the quantitative investigation of synaptic data in other brain regions and species (see Suppl. Notes 6–7 for future directions).

## Methods

### Source dataset

The source dataset for this work was a publicly available collection of synaptic traces and measurements mined from peer-reviewed publications and carefully annotated for detailed metadata as previously described^11^. In this study, we first reconstructed these signals into a set of systematic measurements. Next, we simulated the traces with a synapse model to unify the data format. Then, we created a predictive deep learning model of all the data to infer missing values, disambiguate the identity of presynaptic and postsynaptic neuron types, and normalize the data with respect to covariates. Lastly, we statistically analyzed the resultant completed and normalized dataset and corresponding synaptic simulations.

### Synaptic signal reconstruction

To digitize the mined traces, we used Engauge Digitizer, a multiplatform open-source software (digitizer.sourceforge.net). We implemented a custom Python algorithm, Trace Reconstructor, as part of our Synapse Modeling Utility, to extract a consistent set of data points from each synaptic event, including an initiation, a peak, and a decay point (Suppl. Fig. 2a). Each data point consists of a time and a corresponding amplitude. We found data points either from digitizing traced or through interpolation of reported synaptometric measurements, such as the average amplitude, 10–90% or 20–80% rise times, half-height width (50% rise to 50% decay time), and half-decay time (100%–50% decay time). Six additional intermediate data points were interpolated using the Akima interpolator implemented by SciPy^46^.

For the accurate simulation of ST-P, we ensured all digitized signals had at least 10 successive synaptic events and a recovery event, interpolating them if needed from paired-pulse ratios (PPRs). To infer missing PPRs of facilitating and pseudolinear ST-Ps, we used bicubic interpolation. For depressing signals, we used a custom interpolator that assumed the PPRs exhibit exponential decay to a minimum. For depressing or pseudolinear signals that lacked a recovery event, we assigned a 2s period for recovering from synaptic depression^47–49^. Specifically, we assumed this as the time for the recovery to reach 63% of the difference between the amplitude of the first and the last events in a successive series of events. For facilitating synapses, we did not add a recovery event.

Most synaptic signals start with a fast AMPA or a GABA_A_ response which are gradually mixed with slower synaptic responses or non-synaptic membrane fluctuations. To diminish the impact of slower events, we corrected the signals either at the reconstruction stage or during parametric fitting (Suppl. Fig. 1). When the ISIs of synaptic events were constant, we reconstructed the signal based on the amplitude and the decay time constant of the first synaptic event and the paired-pulse ratios of the successive events. When the ISIs were variable, we used simulated signals to correct the data as described below.

We implemented all the above-mentioned reconstruction algorithms in the Trace Reconstructor tool of the Synapse Modeling Utility.

### Biophysical synaptic model and parametric fitting

To facilitate comparison between current and voltage recordings, we reduced the signals to modality independent synaptic constants utilizing a specific version of the Tsodyks, Pawelzik, and Markram (TPM) model^12,13^. The TPM model formulates a relationship between synaptic conductance (*g*), deactivation time constant (*τ*_d_), recovery time constant (*τ*_r_), facilitation time constant (*τ*_f_), and the utilization ratio (*U*) of synaptic resources in one set of ordinary differential equations. Calculating synaptic currents (*I*_syn_) with an Ohmic model for ion channels (Fig. 3b) requires the reversal potential (*E*_rev_) and the postsynaptic membrane potential (*V*_m_). *E*_rev_ is experimentally measurable or can be accurately estimated from the ionic composition of bath and pipette solutions, temperature, and permeability of ion channels to different ions^11^. We assumed kinetically fast synaptic responses to be mediated by calcium-impermeable AMPA or GABA_A_ channels, unless otherwise stated in the original publications. Because *I*_syn_ is recorded in voltage-clamp experiments, we calculated *V*_m_ by correcting holding potential (*V*_h_) for liquid junction potential (*E*_j_) as previously described^11^.

Using the TPM model, we can analytically simulate the amplitude, kinetics, and ST-P of *I*_syn_. We numerically derived synaptic potentials (*V*_syn_) by feeding the simulated *I*_syn_ to a resistor-capacitor circuit (RC) model of neuronal membrane, from which we equated *V*_syn_ as the evolution of *V*_m_ over time. We used the ODEPACK solver via SciPy for numerical integration. The RC model depends on three experimentally measurable parameters: the membrane time constant (*τ*_m_), membrane capacitance (*C*_m_), and the initial value of *V*_m_. Since *V*_syn_ is recorded in current-clamp experiments, we corrected resting or steady-state membrane potential for E_j_ to estimate the initial value of *V*_m_. We used *τ*_m_ and *C*_m_ values when reported in the original study; otherwise, we utilized the values reported by Hippocampome.org for a matching postsynaptic neuron type in the closest available temperature, recording method, and solutions^10^. If parameters of the RC model could not be found in the original paper or Hippocampome.org, the values were optimized during parametric fitting. Only for 23% (603:2621) of the signals at least one of the *τ*_m_ and C_m_ values was found through optimization.

We found the optimal g, *τ*_d_, *τ*_r_, *τ*_f_, and U values for each experimentally recorded synaptic signal by fitting TPM model simulations to the reconstructed data points. We created a high-performance and user-friendly Python simulator, the Synapse Modeling Utility, to aid in parametric fitting. Optimization was performed by an implementation of the SciPy toolbox genetic algorithm, differential_evolution function, a bound-constrained global optimizer. As the objective function, we chose the mean soft L_1_ squared error, i.e.,

1 $${error}=\frac{2}{n}{\mathop{\sum}\limits _{i=1}^{n}}\left(\sqrt{1+{\left({signa}{l}_{i}-{simulatio}{n}_{i}\right)}^{2}}-1\right)$$

where n is the number of reconstructed data points. We assigned the fitting error associated with the first synaptic event twice the weight relative to all other events, and the 6 interpolated data points half the weight of the initiation, peak, and decay points. We set the following bound constraints: 50 < *τ*_r_ < 3000 ms, 1 < *τ*_f_ < 300 ms, and 0.001 < *U* < 1. Optimization was stopped when the difference of fitting error between successive fits yielded a change of less than 0.001.

If more than one stimulation frequency was available for a given experiment, we pooled data prior to optimization to ensure the estimated parameters are more generalizable to different frequencies. We then re-expanded the data after optimization to match each of the original traces.

The Synapse Modeling Utility also implemented a correction for slow processes when the ISIs varied (see also Supplementary Note 1). In the absence of a slower process, the signal should gradually decay to a baseline. Provided slower processes do not drastically affect the first simulated event such that *τ*_d_ estimation is accurate, the recorded and simulated signals should be most similar at the initiation points of the synaptic events. We defined the correction amounts at initiation points of two successive synaptic events as the amplitude differences (Suppl. Fig. 1b). We then calculated the correction values for data points in between two initiation points by the triangulation method. Signals yielding *τ*_d_ values greater than 700 ms were excluded from subsequent analysis. For signals that only reported amplitude and not kinetics, we set the missing *τ*_d_ values to the median of the unitary GABAergic and glutamatergic responses as appropriate.

Fitting a single synaptic signal using our Synapse Modeling Utility required seconds to minutes, while fitting with a pipeline built using the Neuron Simulation Environment^50^ required hours. We optimized each trace at least 30 times and averaged the best 15 fits. The relative inter-trial variability was <0.001.

### Machine learning design and implementation

We employed machine learning to infer the five parameters of the TPM model (the targets) based on a set of features, namely the pre- and post-synaptic neuron types and covariates. Specifically, the set of features consisted of 319 one-hot encoded and numerical values (Table 1): 122 features encoded presynaptic neuron types, 122 postsynaptic neuron types, and the remaining 75 features encoded the experimental covariates. For instance, three columns encoded four stimulation methods (evoked, unitary, spontaneous, if all three columns were set to zero the stimulation method was miniature), one column encoded three species (1 = rats, −1 = mice, and 0 = guinea pigs), and one column sex (1 = male, −1 = female, and 0 = both or unknown). When the animal age was not reported, we estimated it based on weight, diet, species, and strain. One feature column encoded whether the target signal represented amplitude or potency, which differ in the averaging method: if failed events are excluded from the signal average, the peak quantifies synaptic potency rather than amplitude. For algorithm training, when the failure rate of the first synaptic event was reported, we added an additional pseudo-signal by converting amplitude to/from potency, resulting in a different *g* value as a target and a different potency value as a feature. We normalized features with the MaxAbsScaler function of Scikit-learn toolbox to preserve the sparsity of the feature matrix. Moreover, we normalized the targets with the MinMaxScaler method to allow usage of sigmoid activation functions in the deep learning output layer.

We implemented the machine learning pipeline in Jupyter and Python. We first trained a random forest model using the Scikit-learn package on two Xeon-E5 v3 CPUs to infer missing values of ST-P. The random forest model is a refined series of linear regressions that correct the predictions in every step towards the final output. This algorithm is fully automated, has only one tuning parameter, and is very robust. Specifically, whenever a signal lacked estimates for *τ*_r_, *τ*_f_, and U (typically recordings of single synaptic events), we set those values to zero. However, to allow machine learning to distinguish such ‘not available’ entries from real zero values, we also set the ISI, a feature, to zero in those cases. The random forest model, in contrast to deep learning, could learn to predict missing values for *τ*_r_, *τ*_f_, and U, when ISI was zero. Similarly, the random forest model was able to infer the missing values for these parameters when ISI was non-zero. Specifically, we set missing ISI values to 50 ms, the mode of all ISIs. We then employed the inferred values together with the original values to train a deep learning model utilizing the Keras library with a TensorFlow 2.3 backend on seven NVIDIA Titan X GPUs (Suppl. Fig. 3). First, we trained the deep learning model using the existing experimental data by backpropagating output error. Specifically, during training, information about the target features in the output layer (the TPM parameters) is encoded in the intermediate layers as the information flows backward by back-propagation, and from there all the way to the input layers which represent the features (presynaptic neuron, postsynaptic neuron, and covariates). As a result of this training process, the deep learning model learns to take in the features (presynaptic neuron, postsynaptic neuron, and covariates) and produces a specific target output (five TPM parameter values) based on the input features. The distinction between training features and prediction features in Fig. 3 is that the former ones are linked to the experimentally available traces, whereas the latter ones can be chosen arbitrarily. We also fed back the originally missing values of *τ*_d_, *τ*_r_, *τ*_f_, and *U* estimated by deep learning iteratively until we observed no further improvement in model performance (30 times).

We meticulously hand-tuned the hyperparameters of the deep learning model. Checking different deep learning topologies, we settled on a five-layer autoencoder perceptron, regularized with the latest available techniques to achieve state-of-the-art accuracy and generalization power. Specifically, we used the self-regularized mish activation function^51^, dropout layers^52^ combined with max-norm constraint^53^, batch normalization layers^54^, weight decay regularization^55^, noise regularization^56^, and early stopping technique^57^. As the objective function of the deep learning model, we employed symmetric mean absolute percentage error

2 $${{{{{{\mathrm{SMAPE}}}}}}}=\frac{200}{n}\,{\mathop {\sum} \limits _{i=1}^{n}}\frac{\left|{predictio}{n}_{i}-{targ}{{et}}_{i}\right|}{\left|{predictio}{n}_{i}\right|+\left|{targ}{{et}}_{i}\right|}$$

where *n* is the number of data points^58,59^. It is advantageous to use SMAPE over the competing methods because it is scale-independent and unbiased. We trained the models with the lookahead optimization algorithm^60^ guided by the AdamW optimizer^55^ with weight decay = 0.001, learning rate = 0.015, and batch size = 2621. We implemented learning rate reduction on plateau to achieve the best fit (patience = 100 epochs, factor = 0.9). We used the early stopping technique for restoring the best weights at the end of training to avoid overfitting.

The exact predictions of the model depended on the (randomized) sorting of the training dataset. Thus, we trained 100 models and statistically analyzed the results for each potential connection (Suppl. Fig. 8). The CV of model predictions was not significantly larger in any region and did not correlate with the number of data points available per potential connection. Among parameter predictions, the CV was largest for *τ*_f_, and smallest for *τ*_d_, *U*, and GABAergic *τ*_r_. To maximize robustness, we reported the average value of 100 model inferences for each parameter and potential connection.

The sigmoid activation functions in the output layer ensure model inferences stay data-bound; nevertheless, we also made sure all *g*, *τ*_d_, *τ*_r_, *τ*_f_, and *U* predictions are unique and biologically plausible, i.e., *g* > 0 nS, 0 < *τ*_d_ < 70 ms, *τ*_r_ > 50 ms, *τ*_f_ > 1 ms, and 0 < *U* < 1.

### Machine learning model validation

We computed training accuracy as the average SMAPE distance of the model output from the training data. In contrast, prediction accuracy is the average SMAPE distance of the model output from the unseen data. We monitored the prediction accuracy of the model after each training epoch with k-fold cross-validation^61,62^ with *k* = 4. We trained four models on four separate training runs, each using three-quarters of the data, used the remaining one-fourth of data to measure prediction error, and averaged the results over the four runs. We assessed the final model accuracy with the jackknife method (Fig. 4a, b and Suppl. Fig. 4): we trained *n* = 2621 models with n−1 data points and assessed the prediction error of the model for one set aside data point.

Our dataset had more than one data point for most potential connections (Fig. 2, and Suppl. Fig. 2e, f). In certain cases, these data points had identical features. *Target variability* is the average distance (in SMAPE) of each target value in a group from the group average. For one set of features, predictive models can only predict one set of targets. Therefore, the variability of targets imposes a limit on the maximum accuracy the model can achieve. Considering the average target values as the best estimates of the true values, we calculate the 95% confidence interval around the mean and defined a model prediction as *reliable* if it fell within the confidence interval. Prediction reliability (PR) is the percentage of model predictions that are within the confidence interval.

### Data normalization

The training features were highly heterogeneous and typically mapped to multiple presynaptic and/or postsynaptic neuronal types (fuzzy or ambiguous mapping). Nevertheless, a trained model can predict targets (synaptic parameters) for an arbitrary set of features. We inferred values for the unambiguous (proper) mapping of all 3120 potential connections in the entorhinal-hippocampal network. In other words, each inference feature was mapped to one presynaptic neuron type and one postsynaptic neuron type. We also set all other features except the presynaptic and postsynaptic neuronal types to identical values. For instance, we selected identical ionic concentrations for physiological solutions across all synapses and calculated *E*_rev_ accordingly. We set no NMDA or GABA_B_ contamination for the features. Using the trained deep learning models, we inferred unitary synaptic parameters for each potential connection always verifying that the predicted values remained within the upper and lower boundaries of the training set to avoid erroneous extrapolations. We chose unitary postsynaptic currents recorded from adult male rat slices kept in artificial cerebrospinal fluid at body temperature while using whole-cell patch pipettes devoid of high [Cl]_i_ or [gluconate]_i_ solutions as a standard condition for model inferences. When analyzing covariates, we changed one feature at a time to infer the corresponding synaptic parameter. We also generated the inferences for 32 different permutations of conditions, i.e., rat vs mice, male vs female, P14 (adolescent) vs P56 (adult), room (22 °C) vs body (32 °C) temperatures, and voltage-clamp vs current-clamp recording methods.

### Statistics and reproducibility

To compare synapses, we either analyzed the TPM model parameters directly or simulated each synapse separately and measured different synaptometrics. The paired-pulse ratio is the measure of ST-P, which requires the estimation of amplitude. For the first synaptic event, the baseline crosses the initiation points, but for later events, the overlap of initiation points with the baseline depends on the ISI and *τ*_d_ values. If the amplitude is measured from the baseline, we used the *AB*_*i*_ term, where *i* is the event number. Otherwise, if the amplitude is measured from the initiation point, we used the *A*_*i*_ term. For example, *A*_*1*_ is the amplitude of the first synaptic event, and *AB*_*3*_ is the amplitude from the baseline of the third synaptic event (Figs. 3 and 7). Thus, *AB*_*i*_*:A*_*1*_ represents the paired-pulse ratio of the ith event from baseline, which assesses the evolution of the synaptic activation (see derivation of TPM model section for mathematical clarification).

We compared groups with paired or unpaired Wilcoxon’s test as appropriate. We corrected all p-values for multiple comparisons using False Discovery Rate^63^ and selected 0.05 as the significance threshold. We corrected coefficients of variation (CVs) for sample sizes and used the bootstrapping method to find the confidence intervals^64,65^. We used the Pearson method to compute correlations. The p-value of correlations is calculated using t distribution table and ggpubr package in R.

As a measure of central tendency, we defined trimmed-mean as a mean value in which 2.5% of outliers on both extremes are excluded. The interquartile range is the measure of spread. Since the synaptic parameters have different units, we used symmetric percentage distance

3 $${{{\mathrm{SPD}}}}=200\frac{{v}_{1}-{v}_{2}}{\left|{v}_{1}\right|+\left|{v}_{2}\right|}$$

as a measure of change between two data points that is dimensionless and unbiased. We simply use the term percentage to refer to SPD in the Results and Discussion of this paper. Specifically, for covariates analysis (Fig. 6b), we computed the trimmed-mean of SPDs of reference vs change of each potential connection:

4 $${{{\mathrm{SPD}}}}=200\frac{{change}-{reference}}{\left|{change}\right|+\left|{reference}\right|}$$

Then, compared the differences with paired Wilcoxon’s test. For morphology and marker analysis (Fig. 8b, c), we used the unpaired Wilcoxon’s test. We converted the Wilcoxon’s estimate, a robust measure of the difference between groups, and 95% confidence intervals to SPD by multiplying these values by

5 $$\frac{200}{{median}\left(\left(-\right)\right)+{median}\left(\left(+\right)\right)}$$

where (+) refers to the group that expressed the marker and (−) for the group that did not.

### Derivation of a simplified Tsodyks, Pawelzik and Markram synapse model

Over the last 50 years, a large body of phenomenological synaptic plasticity models has been theorized^12^. One of the better-established models is that of Tsodyks, Pawelzik, and Markram (TPM)^13^. In this work, we adapted a simplified version of the TPM model^12^ and further streamlined the analytical solutions.

### Ordinary differential equations describing synaptic temporal dynamics

Short-term synaptic plasticity depends on the availability and utilization of synaptic resources (Fig. 3b), including the number of readily releasable synaptic vesicles and the concentration of calcium. Short-term synaptic facilitation begins with an increase of calcium ions within the presynaptic terminal resulting in an increase in synaptic resource utilization. Equation 6 formulates the utilization dynamics:

6 $$\frac{{du}}{{dt}}=-\frac{u}{{\tau }_{f}}+U\cdot \left(1-{u}_{-}\right)\cdot \delta \left(\varDelta {t}_{i}\right)\;\;\;\;{with}\;\;\varDelta {t}_{i}={t-t}_{i}$$

where $u$ is the fractional degree of synaptic utilization at any moment $t$, ${u}_{-}$ indicates the value of $u$ just before the synaptic event time ${t}_{i}$, $U$ determines the increment proportion (between 0 and 1) with each presynaptic spike, and $\delta$ is Dirac’s delta function. Since ($1-u$) quantifies unutilized resources and synapses cannot use more than all the resources available to them, $U\cdot \left(1-{u}_{-}\right)$ determines $u$ increment after each synaptic event. Whenever a synapse is not being stimulated, synaptic utilization exponentially decays to zero with the facilitation decay time constant ${\tau }_{f}$.

Synaptic depression is due to the depletion of available synaptic resources. These resources can be partitioned into three portions, representing respectively the activated (A), deactivated (D), and recovered (R) states. After each presynaptic spike, an instantaneous shift occurs from recovered to activated state. The amount of shift is determined by $u$. The active resources then decay to the deactivated state by the decay time constant ${\tau }_{d}$. Since synaptic resources are limited, the more resources stay in the deactivated state, the more a synapse is depressed. In the TPM model, synaptic resources exponentially recover from depression with the recovery time constant *τ*_*r*_. This process can be formulated by the following set of equations:

7 $$\frac{{dR}}{{dt}}=\,\frac{D}{{\tau }_{r}}-{u}_{+}{R}_{-}\delta \left(\varDelta {t}_{i}\right)$$

8 $$\frac{{dA}}{{dt}}=\,-\frac{A}{{\tau }_{d}}+{u}_{+}{R}_{-}\delta \left(\varDelta {t}_{i}\right)$$

9 $$\frac{{dD}}{{dt}}=\,\frac{A}{{\tau }_{d}}-\frac{D}{{\tau }_{r}}$$

where ${u}_{+}$ is the value of $u$ just after synaptic event time, which can be determined using Eq. 6. ${R}_{-}$ is the value of *R* just before the synaptic event, which is determined by Eq. 7. The product ${u}_{+}{R}_{-}$ represents the fraction of the synaptic resources being utilized after each synaptic event. This proportion is added to the already active resources ($A$) and taken from the readily usable resources ($R$). Then, the change in $D$ at any moment is the difference between resources deactivating ($\frac{A}{{\tau }_{d}}$) and resources recovering ($\frac{D}{{\tau }_{r}}$).

A simplified version of the four-state TPM model^12^ eliminated Eq. 9 which is possible since the total amount of synaptic resources is fixed:

10 $$R\,+\,A\,+\,D\,=1$$

Substituting $D=1-R-A$, the four-state TPM model can be reduced to the following three-state model:

11 $$\left\{\begin{array}{c}\frac{du}{dt}=-\frac{u}{\tau_{f}}+U\cdot (1-{u}_{-})\delta (\varDelta {t}_{i})\hfill\\ \frac{dR}{dt}=\frac{1-R-A}{\tau_{r}}-{u}_{+}{R}_{-}\delta (\varDelta{t}_{i})\hfill\\ \frac{dA}{dt}=-\frac{A}{\tau_{d}}+{u}_{+}{R}_{-}\delta (\varDelta {t}_{i})\hfill\end{array}\right.$$

### Analytical solution of the model

This three-state model can be solved using the technique of exact integration^66^. If $\frac{{df}}{{dt}}$ is a time varying function of S(t),

$$\frac{{df}}{{dt}}=\frac{1}{\tau }\left(-f+S\left(t\right)\right)$$

$$f\left(t\right)$$ is:

12 $$f\left(t\right)={f}_{{t}_{i-1}}{e}^{-\frac{\triangle {t}_{i-1}}{\tau }}+\frac{1}{\tau }{e}^{-\frac{t}{\tau }}\int _{{t}_{i-1}}^{t}{e}^{\frac{{t}^{{\prime} }}{\tau }}S\left({t}^{{\prime} }\right)\,d{t}^{{\prime} }$$

Applying this formula to solve Eq. 6:

$$u\left(t\right)={u}_{{t}_{i-1}}{e}^{-\frac{\triangle {t}_{i-1}}{{\tau }_{f}}}+U\left(1-{u}_{-}\right){e}^{-\frac{t}{{\tau }_{f}}}\int _{{t}_{i-1}}^{t}{e}^{\frac{{t}^{{\prime} }}{{\tau }_{f}}}\delta \left(\triangle {t}_{i}^{{\prime} }\right)d{t}^{{\prime} }$$

Since ${\int }_{-\infty }^{\infty }\;\,f\left(t\right)\delta \left(t\right)=f\left(0\right)$, we will have:

13 $$u\left(t\right)=\left\{\begin{array}{cl}{u}_{{t}_{i-1}}{e}^{-\frac{\triangle {t}_{i-1}}{{\tau }_{f}}}\hfill & {if}{\,} t{\,}\ne {\,}{t}_{i}\\ {u}_{-}+U\left(1-{u}_{-}\right) & {if}{\,} t={t}_{i}\end{array}\right.$$

Similarly, the solution for $A\left(t\right)$ is:

$$A\left(t\right)={A}_{{t}_{i-1}}{e}^{-\frac{\triangle {t}_{i-1}}{{\tau }_{d}}}+{u}_{+}{R}_{-}{e}^{-\frac{t}{{\tau }_{d}}}\int _{{t}_{i-1}}^{t}{e}^{\frac{{t}^{{\prime} }}{{\tau }_{d}}}\delta \left(\triangle {t}_{i}^{{\prime} }\right)d{t}^{{\prime} }$$

14 $$A\left(t\right)=\left\{\begin{array}{cl}{A}_{{t}_{i-1}}{e}^{-\frac{\triangle {t}_{i-1}}{{\tau }_{d}}}\hfill & {if}{\,} t{\,}\ne {\,}{t}_{i} \\ {A}_{-}+{u}_{+}{R}_{-} & {if}{\,} t={t}_{i}\end{array}\right.$$

The solution for $R\left(t\right)$ is:

$$R\left(t\right)={R}_{{t}_{i-1}}{e}^{-\frac{\triangle {t}_{i-1}}{{\tau }_{r}}}+\frac{1}{{\tau }_{r}}{e}^{-\frac{t}{{\tau }_{r}}}\int _{{t}_{i-1}}^{t}{e}^{\frac{{t}^{{\prime} }}{{\tau }_{r}}}\left(1-A\left({t}^{{\prime} }\right)-{\tau }_{r}{u}_{+}{R}_{-}\delta \left(\triangle {t}_{i}^{{\prime} }\right)\right){{dt}}^{{\prime} }$$

Substituting A from Eq. 14 and expanding the integral, yields:

$$R\left(t\right)=	\; {R}_{{t}_{i-1}}{e}^{-\frac{\triangle {t}_{i-1}}{{\tau }_{r}}}+\frac{1}{{\tau }_{r}}{e}^{-\frac{t}{{\tau }_{r}}}\left(\int _{{t}_{i-1}}^{t}{e}^{\frac{{t}^{{\prime} }}{{\tau }_{r}}}d{t}^{{\prime} }-{A}_{{t}_{i-1}}\int _{{t}_{i-1}}^{t}{e}^{\frac{{t}^{{\prime} }}{{\tau }_{r}}-\frac{\triangle {t}_{i-1}^{{\prime} }}{{\tau }_{d}}}d{t}^{{\prime} }\right.\\ 	 \left.-{\tau }_{r}{u}_{+}{R}_{-}\int _{{t}_{i-1}}^{t}{e}^{\frac{{t}^{{\prime} }}{{\tau }_{r}}}\delta \left(\triangle {t}_{i}^{{\prime} }\right)d{t}^{{\prime} }\right)$$

Since $${\int }_{a}^{b}\ f\left(t\right){dt}=F\left(b\right)-F\left(a\right)$$,

$$R\left(t\right)=	\; {R}_{{t}_{i-1}}{e}^{-\frac{\triangle {t}_{i-1}}{{\tau }_{r}}}+\frac{1}{{\tau }_{r}}{e}^{-\frac{t}{{\tau }_{r}}}\left({\tau }_{r}\left({e}^{\frac{t}{{\tau }_{r}}}-{e}^{\frac{{t}_{i-1}}{{\tau }_{r}}}\right)-\frac{{A}_{{t}_{i-1}}{\tau }_{r}{\tau }_{d}}{{\tau }_{d}-{\tau }_{r}}{e}^{\frac{{t}_{i-1}}{{\tau }_{d}}}\left({e}^{\frac{t}{{\tau }_{r}}-\frac{t}{{\tau }_{d}}}-{e}^{\frac{{t}_{i-1}}{{\tau }_{r}}-\frac{{t}_{i-1}}{{\tau }_{d}}}\right)\right)\\ 	 -{u}_{+}{R}_{-}\int _{{t}_{i-1}}^{t}{e}^{-\frac{\triangle {t}_{i-1}}{{\tau }_{r}}}\delta \left(\triangle {t}_{i}^{{\prime} }\right)d{t}^{{\prime} }$$

Which simplifies to the following equation assuming ${\bar{A}}_{{t_{i-1}}}=\frac{{A}_{{t_{i-1}}}{\tau }_{d}}{{\tau }_{d}-{\tau }_{r}}$:

15 $$R(t)=\left\{\begin{array}{cl}1-{\bar{A}}_{{t}_{i-1}}{e}^{-\frac{\varDelta{{t}_{i-1}}}{\tau_{d}}}-(1-R_{{t}_{i-1}}-{\bar{A}}_{{t}_{i-1}}){e}^{-\frac{\varDelta{{t}_{i-1}}}{\tau_{r}}} & {if}{\;}t {\,}\ne {\,}{t}_{i}\hfill\\ {R}_{-}-{u}_{+}{R}_{-}\hfill & if {\;}t={t}_{i}\end{array}\right.$$

### Summary of the analytical solution

Since $A\left(t\right)$ is independent of the rest of the equations, the simulation of synaptic amplitude after each synaptic event only requires the calculation of Eq. 14. When a synaptic event occurs, the value of each of the states should be calculated just before the synaptic event.

16 $$\left\{\begin{array}{c}{u}_{-}={u}_{{t_{i-1}}}{e}^{-\frac{\varDelta {{t_{i-1}}}}{{\tau }_{f}}}\hfill \\ {A}_{-}={A}_{{t_{i-1}}}{e}^{-\frac{\varDelta {{t_{i-1}}}}{{\tau }_{d}}}\hfill \\ {R}_{-}=1-\frac{{\bar{A}}_{{t_{i-1}}}{A}_{-}}{{A}_{{t_{i-1}}}}-(1-{R}_{{t_{i-1}}}-{\bar{A}}_{{t_{i-1}}}){e}^{-\frac{\varDelta {t_{i-1}}}{{\tau }_{r}}}\end{array}\right.$$

Note that only three exponential function evaluations are required if ${A}_{-}$ is calculated just before the calculation of ${R}_{-}$. Once pre-event values have been calculated, the following set of equations are used to update $u$, $A$, and $R$:

17 $$\left\{\begin{array}{c}{u}_{{t}_{i-1}}={u}_{+}={u}_{-}\,+\,U\,\left(1\,-\,{u}_{-}\right)\\ {A}_{{t_{i-1}}}={A}_{+}={A}_{-}+\,{u}_{+}{R}_{-}\hfill\\ {R}_{{t_{i-1}}}={{R}_{+}=R}_{-}-\,{u}_{+}{R}_{-}\hfill\end{array}\right.$$

We emphasize that the order of equations is important: since ${u}_{+}$ is the value of u just after a synaptic event, A and R must be updated after ${u}_{+}$.

### The first synaptic event

Ohm’s law is used to calculate the synaptic currents (${I}_{{syn}}$):

$${I}_{{syn}}=g\times \left({V}_{m}-{E}_{{rev}}\right)$$

In the TPM model, ${I}_{{syn}}$ is calculated with the following equation:

$${I}_{{syn}}={g}_{{optimization}}\times A\times \left({V}_{m}-{E}_{{rev}}\right)$$

Therefore,

$$g={g}_{{optimization}}\times A$$

Before any synaptic event, all resources are readily usable, and there is no utilization and activation. Therefore,

$$\left\{\begin{array}{c}{u}_{{t}_{0}}=0\\ {A}_{{t}_{0}}=0\\ {R}_{{t}_{0}}=1\hfill\end{array}\right.$$

For the first synaptic event, the value of *A* is easily calculatable.

$$\left\{\begin{array}{c}{u}_{{t}_{1}}={u}_{{t}_{0}}\,+\,U\,\left(1\,-\,{u}_{{t}_{0}}\right)=U\\ {A}_{{t}_{1}}={A}_{{t}_{0}}+\,{u}_{{t}_{1}}{R}_{{t}_{0}}=U\hfill\end{array}\right.$$

Therefore,

18 $$g={g}_{{optimization}}\times {A}_{{t}_{1}}={g}_{{optimization}}\times U$$

### Distinction between short-term plasticity measures

The distinction between the paired-pulse ratio (${PP}{R}_{i:1}$) and paired-pulse ratio from the baseline $\left(A{B}_{i}:{A}_{1}\right)$ is formulated with the following equations:

19 $${PP}{R}_{i:1}=\frac{{A}_{{t}_{{i}_{+}}}-{A}_{{t}_{{i}_{-}}}}{{A}_{{t}_{{1}_{+}}}-{A}_{{t}_{{1}_{-}}}}=\frac{{u}_{{{t}_{i}}_{+}}{R}_{{t}_{{i}_{-}}}}{{u}_{{{t}_{1}}_{+}}{R}_{{t}_{{1}_{-}}}}$$

20 $$A{B}_{i}:{A}_{1}=\frac{{A}_{{t}_{{i}_{+}}}}{{A}_{{{t}_{1}}_{+}}}$$

These equations indicate that the $A{B}_{i}:{A}_{1}$ measures the evolution of A state but ${PP}{R}_{i:1}$the evolution of ${u}_{+}{R}_{-}$.

### Convergence of numerical and analytical solutions

We implemented the numerical and analytical solutions in the NEURON simulation environment^50^ and compared them to the original four-state model to confirm the convergence of all the formalisms (Suppl. Fig. 9). The simulation files are available to download from the ModelDB portal (Accession: 266934).

### Reporting summary

Further information on research design is available in the Nature Research Reporting Summary linked to this article.

## Supplementary information

Supplementary Information

Description of Additional Supplementary Files

Supplementary Data 1

Supplementary Data 2

Supplementary Movie 1

Reporting Summary

## Data Availability

Supplementary Data 1 provides the source data underlying Figs. 6a and 8. All other data are released on Hippocampome.org/synapse. The underlying experimental measurements come from Hippocampome.org/synaptome as described in^11^. Supplementary Data 2 provides the list of all 160 articles reporting those measurements.

## Code availability

The Synapse Modeling Utility developed in this work is available at https://github.com/k1moradi/SynapseModelingUtility, 10.5281/zenodo.6385650, and https://hippocampome.org/general/synapse_modeling/Modeling.rar. The Machine Learning Library is available at https://github.com/k1moradi/MachineLearningSynapsePhysiology, 10.5281/zenodo.6385648, and https://hippocampome.org/general/synapse_modeling/MachineLearningSynapsePhysiology.zip.
