# Supplementary material for: Normalized unitary synaptic signaling of the hippocampus and entorhinal cortex predicted by deep learning of experimental recordings
Source: Commun Biol. 2022 May 5;5:418. doi: 10.1038/s42003-022-03329-5 (PMC9072429; doi:10.1038/s42003-022-03329-5)
Supplement: Supplementary file 1 — Supplementary Information [file 42003_2022_3329_MOESM1_ESM.pdf]

Normalized unitary synaptic signaling of the hippocampus and entorhinal cortex predicted by deep learning of experimental recordings

Keivan Moradi, Zainab Aldarraji, Megha Luthra, Grey Madison, Giorgio A. Ascoli

Supplementary Figures

Supplementary Figure 1: Signal correction methods and analysis of genetic algorithm optimization error.

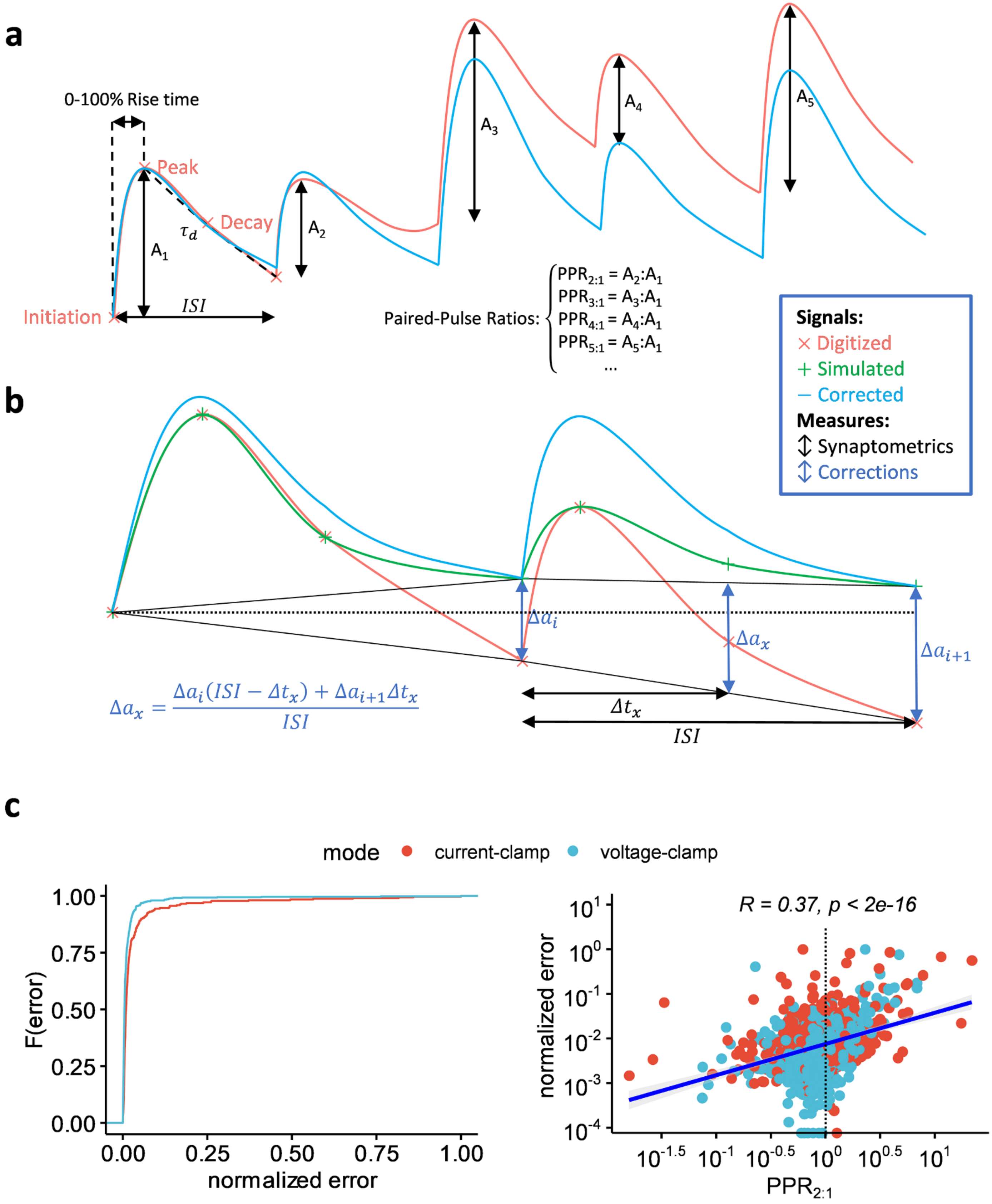

We removed any membrane fluctuations with slow kinetics superposed on the recorded synaptic signals using two methods. **(a)** We extracted the amplitude (A) and deactivation time constant (τ_d) of the first synaptic event from a digitized trace to reconstruct the signal by estimating the initiation points. **(b)** We used a simulated signal to approximate the correction amount at the initiation points of synaptic events (e.g., Δa_i and Δa_{i+1}). Triangulation was then used to linearly approximate the correction amounts of intermediate points (Δa_x). **(c)** For the analysis of the optimization error, we made a database of all the soft L1 loss values, and grouped the data based on recording mode. We then normalized the error by subtracting the minimum and dividing by the maximum in each group. The cumulative density of normalized error indicates that the optimization error is minimal for the majority of cases, suggesting the simulated signal satisfactory fits the experimental recording. In addition, the normalized error had a positive correlation with the paired pulse ratio of the second synaptic event compared with the first ($PPR_{2:1}$) suggesting short-term depression compared with short-term facilitation is simulated more accurately by the TPM model.

Supplementary Figure 2: Impact of recording and stimulation methods on synaptic properties and data availability.

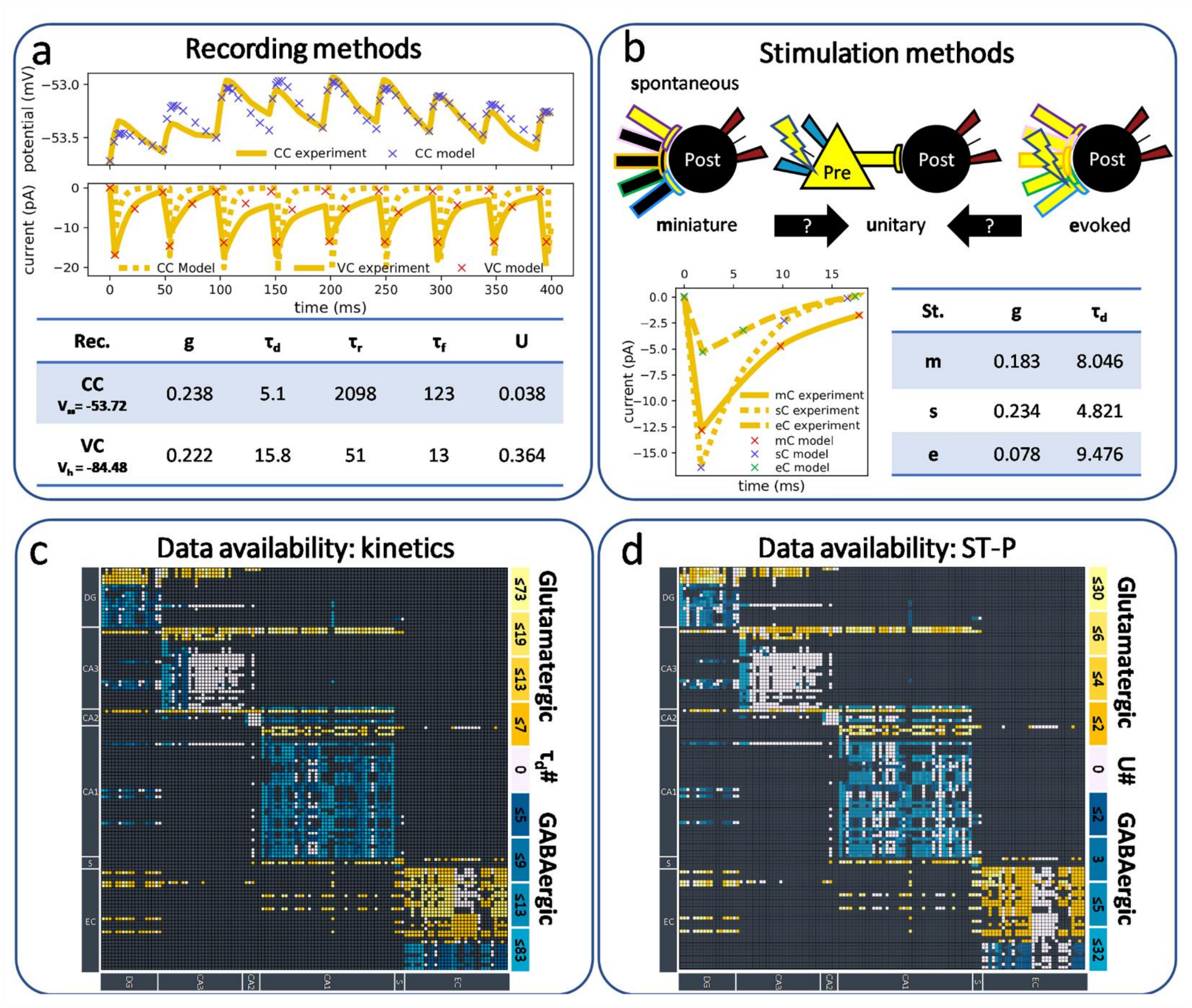

(a) Voltage- and current-clamp recording of glutamatergic synaptic signals between CA3 Pyramidal cells at two different membrane potentials[1]. Even though the estimated synaptic conductances are almost equal, the rest of the parameter estimates differ substantially. **(b)** Glutamatergic synaptic currents recorded from CA1 Basket CCK+ neurons using three different stimulation paradigms[2]. Evoked currents were unexpectedly smaller than miniature and spontaneous ones, because of the minimal stimulation protocol. Note that researchers only reported one synaptic event, preventing the estimation of τ_r, τ_f, and U. **(c-d)** Heatmap representations of the number of data points available for each of 3,120 potential connections among 122 neuron types (rows: presynaptic, columns: postsynaptic). Light pink entries are entries with missing synaptic data (19.7% for τ_d and 38.5% for ST-P parameters). Black entries mark absence of potential connection. In all panels (a-f), blue and yellow colors represent GABAergic and glutamatergic synapses, respectively.

Supplementary Figure 3: Deep learning model architecture.

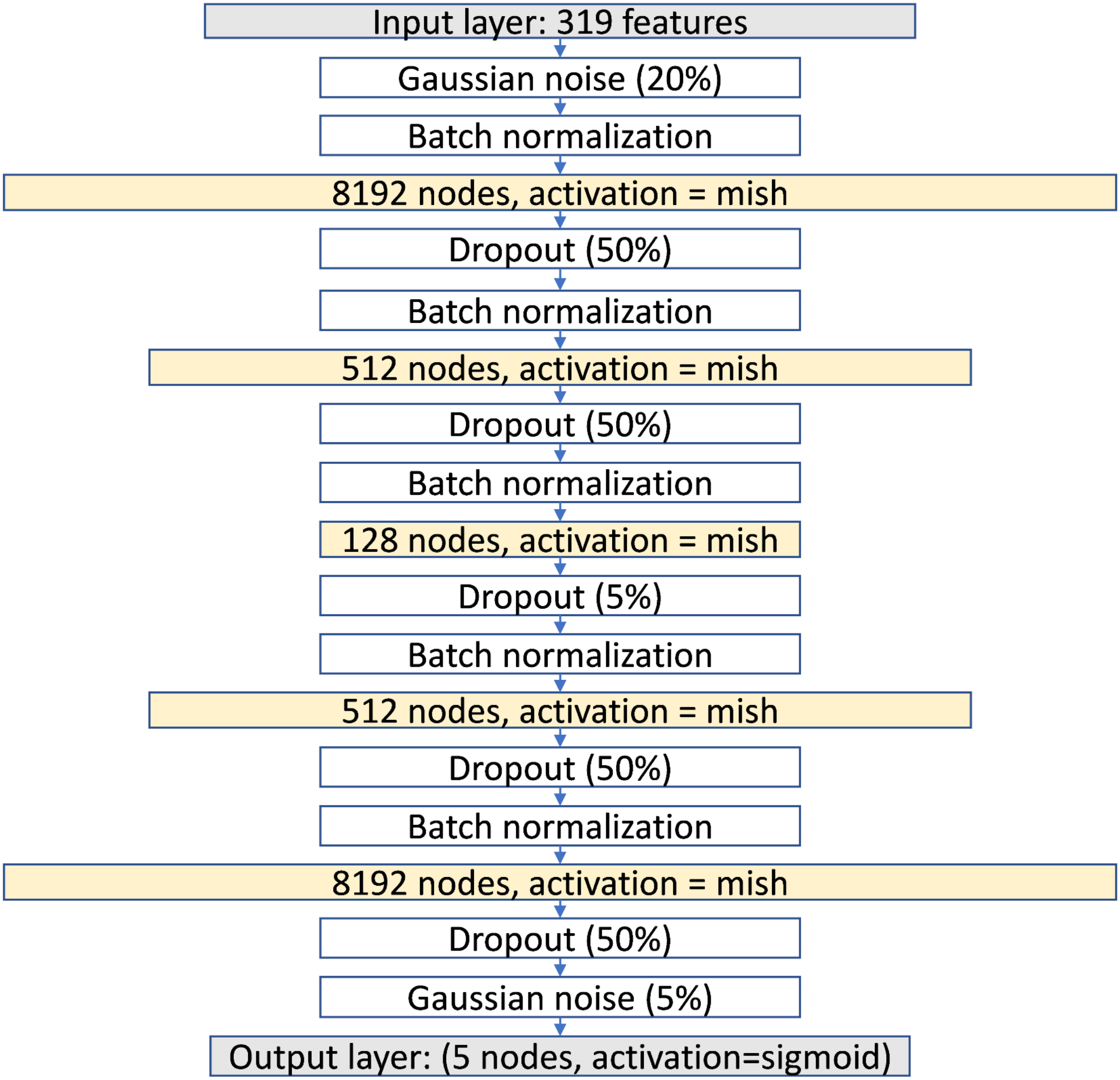

The deep learning model in this study is a five-layer encoder-decoder (autoencoder) perceptron regularized with different techniques to prevent overfitting and maximize the prediction power. In this architecture, the inputs (features) are converted to a series of internal representations (encoding) until they reach the layer with the smallest size (encoder layer). Then, the internal representation is progressively translated by the remaining layers (decoding) eventually resulting in the outputs (predictions). We attained the best results with 128 nodes in the encoder (third) layer. For model regularization, we set a 50% dropout rate for all layers

except for the encoder layer, which was set to 5%. Prediction accuracy improved by coupling dropout with unitary max-norm weight constraint and batch normalization.

Supplementary Figure 4: Performance of the deep learning model for all types of stimulation methods.

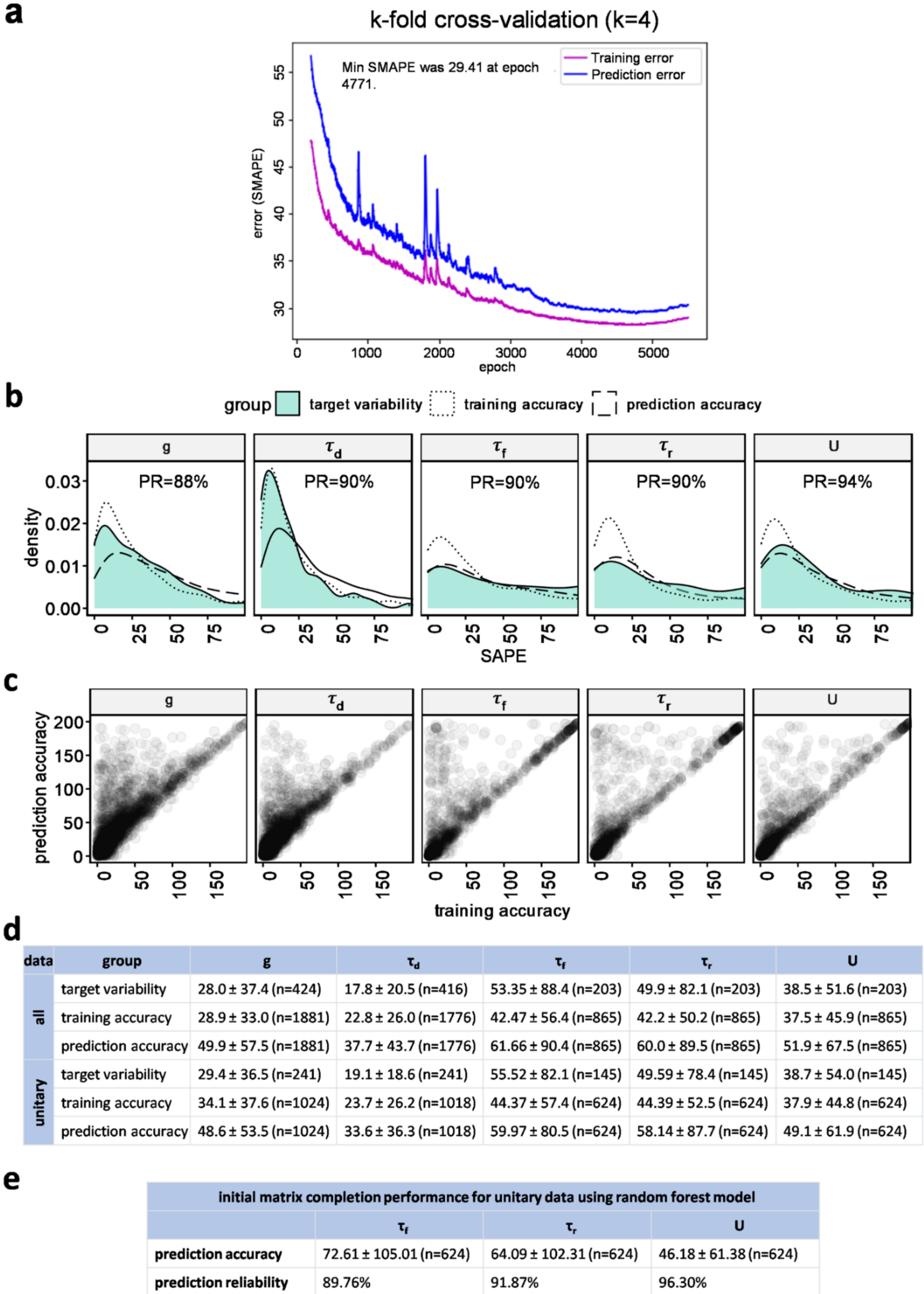

d

data	group	g	τ_d	τ_f	τ_r	U
all	target variability	28.0 ± 37.4 (n=424)	17.8 ± 20.5 (n=416)	53.35 ± 88.4 (n=203)	49.9 ± 82.1 (n=203)	38.5 ± 51.6 (n=203)
	training accuracy	28.9 ± 33.0 (n=1881)	22.8 ± 26.0 (n=1776)	42.47 ± 56.4 (n=865)	42.2 ± 50.2 (n=865)	37.5 ± 45.9 (n=865)
	prediction accuracy	49.9 ± 57.5 (n=1881)	37.7 ± 43.7 (n=1776)	61.66 ± 90.4 (n=865)	60.0 ± 89.5 (n=865)	51.9 ± 67.5 (n=865)
unitary	target variability	29.4 ± 36.5 (n=241)	19.1 ± 18.6 (n=241)	55.52 ± 82.1 (n=145)	49.59 ± 78.4 (n=145)	38.7 ± 54.0 (n=145)
	training accuracy	34.1 ± 37.6 (n=1024)	23.7 ± 26.2 (n=1018)	44.37 ± 57.4 (n=624)	44.39 ± 52.5 (n=624)	37.9 ± 44.8 (n=624)
	prediction accuracy	48.6 ± 53.5 (n=1024)	33.6 ± 36.3 (n=1018)	59.97 ± 80.5 (n=624)	58.14 ± 87.7 (n=624)	49.1 ± 61.9 (n=624)

e

initial matrix completion performance for unitary data using random forest model			
	τ_f	τ_r	U
**prediction accuracy**	72.61 ± 105.01 (n=624)	64.09 ± 102.31 (n=624)	46.18 ± 61.38 (n=624)
**prediction reliability**	89.76%	91.87%	96.30%

(a) Prediction error of the model monitored after each training epoch using k-fold cross-validation. The prediction error was close to the training error and the difference between the two decreased after each training epoch. **(b-c)** Comparison of training and prediction accuracies with target variability for all types of synaptic stimulations. These results, including prediction reliability (PR), are comparable to those obtained when only considering unitary stimulation (Fig. 4). **(d)** Trimmed-mean and interquartile range of target variability and training and prediction accuracies (in SMAPE) for different parameters. **(e)** The prediction accuracy and reliability of the initial random forest model in inferring short-term plasticity parameters (ST-P) as determined with jackknife method. From the subset of training data for which ST-P parameters were available, we removed the values of τ_r, τ_r, and U for one data point at a time. We then let the random forest model predict the missing ST-P parameters. The resulting accuracy was comparable with the prediction accuracy of the final deep learning model. In cases multiple measurements were available, predictions were reliable 90% of the times.

Supplementary Figure 5: Model validation.

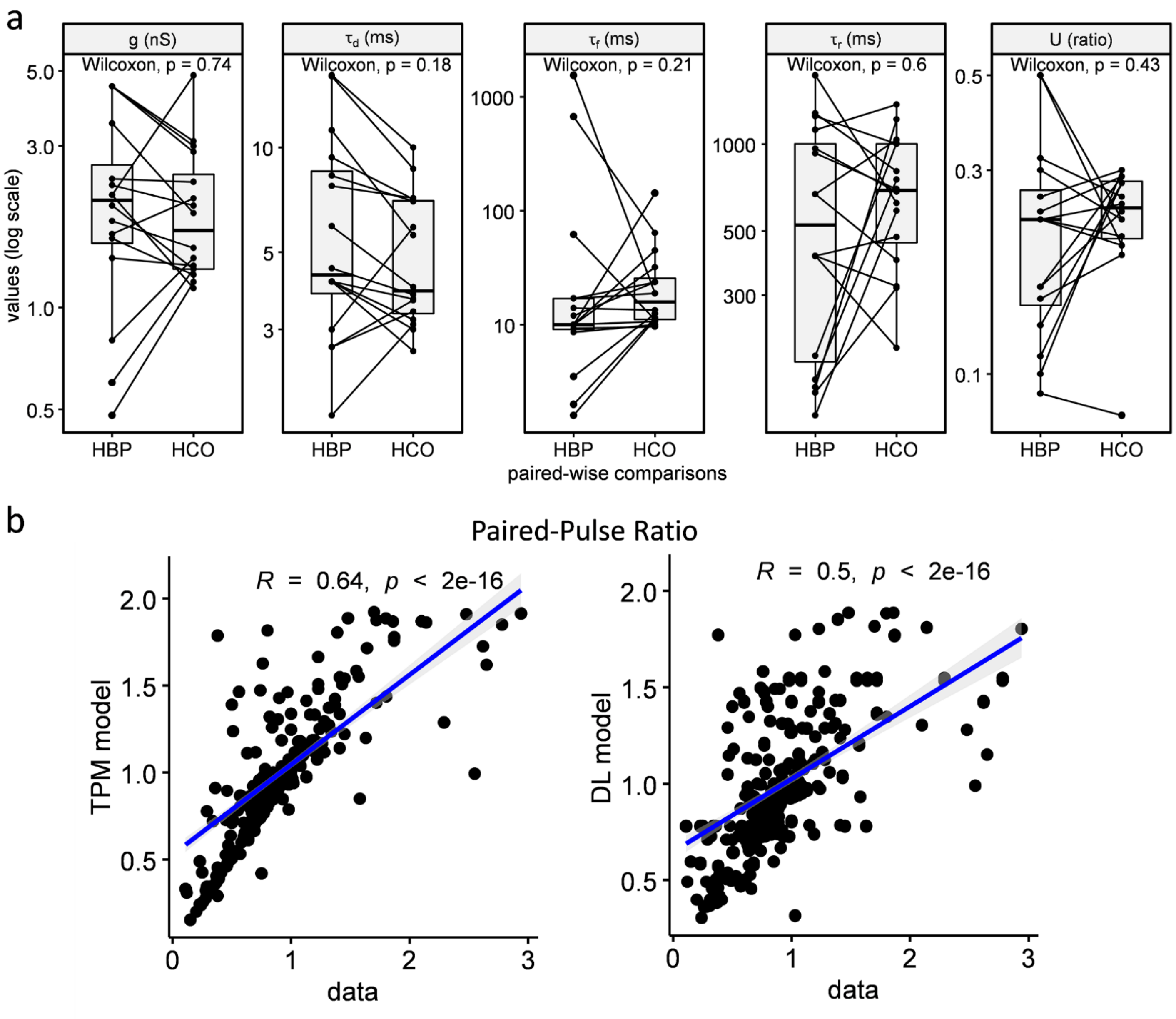

(a) Comparison of synaptic parameters with existing sparse estimates in CA1. The human brain project (HBP) has recently estimated synaptic parameters of 16 potential connections in CA1[3]. A pairwise comparison by potential connections and synaptic parameter with our estimates to be included in Hippocampome.org (HCO) detects no statistically significant difference. The lower and upper hinges of the box and whiskers plot correspond to the first and third quartiles around median. **(b)** The correlation of paired-pulse ratio ($PPR_{2:1}$) of simulated signal and experimental unitary current recordings. $PPR_{2:1}$ of the simulated signal (TPM model) is correlated with the data just after parametric fitting stage. $PPR_{2:1}$ remains correlated with $PPR_{2:1}$ of the experimental recordings after deep learning (DL model), which corroborates that the deep learning model produces valid results. The gray shade next to regression line represents the 95% confidence interval.

Supplementary Figure 6: Range and distribution of synaptic parameters in different anatomical regions.

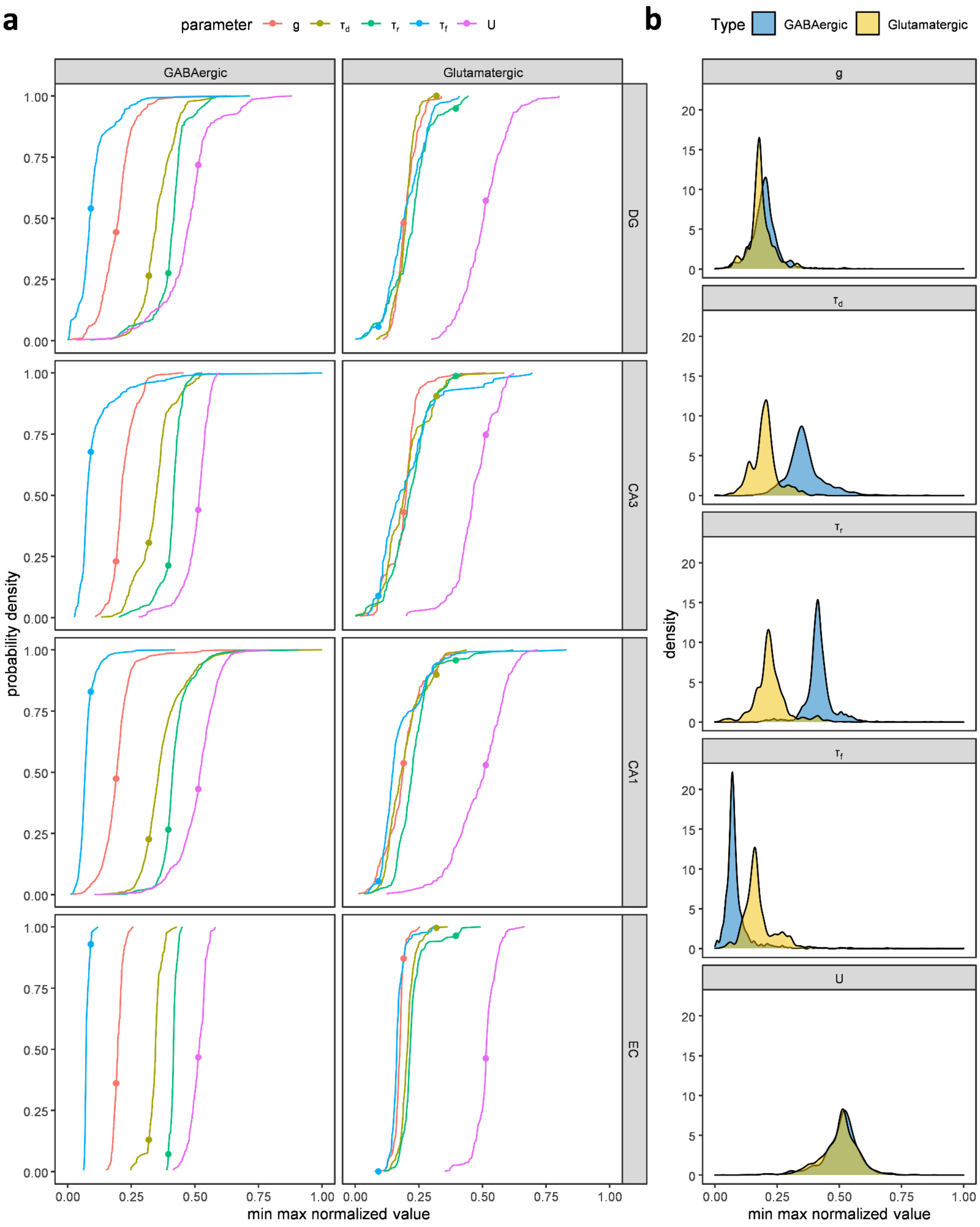
a
parameter
g
τd
τr
τf
U
GABAergic
Glutamatergic
DG
CA3
CA1
EC
probability density
1.00
0.75
0.50
0.25
0.00
0.00
0.25
0.50
0.75
1.00
min max normalized value
b
Type
GABAergic
Glutamatergic
g
τd
τr
τf
U
density
20
15
10
5
0
min max normalized value

(a) Probability density functions of the synaptic parameter in standard conditions globally normalized with the min-max method across different regions and synapse types. Filled circles denote median parameter values.
(b) Distributions of min-max normalized GABAergic and glutamatergic parameter inferences. All synaptic parameters except U are right-tailed. The three time constants, but not g and U, differ by neurotransmitter: relative to GABAergic, glutamatergic synapses have smaller τ_d and τ_r, but larger τ_f.

Supplementary Figure 7: Influence of axonal targeting patterns on synaptic input.

a

Region	Axons		GABAergic					Glutamatergic				
	G_1	G_2	g	τ_d	τ_r	τ_f	U	g	τ_d	τ_r	τ_f	U
CA1	OR	OPR	**17.6**	**-15.6**	-0.2	-9.2	4.1	4.2	-2.5	2.2	0.6	0.8
	SLM	OPR	**-11.2**	**-10.0**	-0.2	**16.8**	**-21.2**	-11.0	-5.7	-5.2	17.9	-22.5
	SLM	OR	**-28.4**	6.3	0.0	**26.0**	**-25.0**	-18.3	-2.0	-7.4	16.7	-22.1
	SP	OPR	-1.3	**-16.7**	-0.4	**25.1**	4.7	-7.2	-12.5	-3.5	**26.7**	9.6
	SP	OR	**-19.1**	-3.1	0.1	**34.2**	0.3	-11.8	-9.0	-5.1	23.3	10.5
	SP	SLM	8.7	-6.0	0.1	10.0	**25.3**	1.5	-7.9	1.4	6.0	**30.6**
CA2	SP	OPR	-23.1	5.3	**51.4**	11.8	-72.7	0.8	5.6	121	-19.9	-68.5
	SP	SR	-7.6	15.8	19.7	-1.5	-73.2	31.5	11.7	1.8	-1.6	-67.7
	SR	OPR	-17.9	-10.8	31.2	14.3	0.3	-25.2	-3.8	89.4	-38.2	-2.7
CA3	SLM	OPR	-4.3	6.7	-8.6	**16.9**	-0.1	-1.5	9.3	-13.9	23.1	-1.7
	SP	OPR	0.3	**-10.1**	0.3	-4.2	2.3	-4.1	-11.1	12.4	-15.5	2.6
	SP	SLM	5.1	**-19.4**	9.4	**-24.2**	2.7	-0.9	**-21.6**	**27.2**	**-41.2**	5.0
DG	SG	SMo	-1.8	-8.2	**-26.5**	-13.1	1.8	1.6	**-13.0**	-43.9	11.9	8.8

b

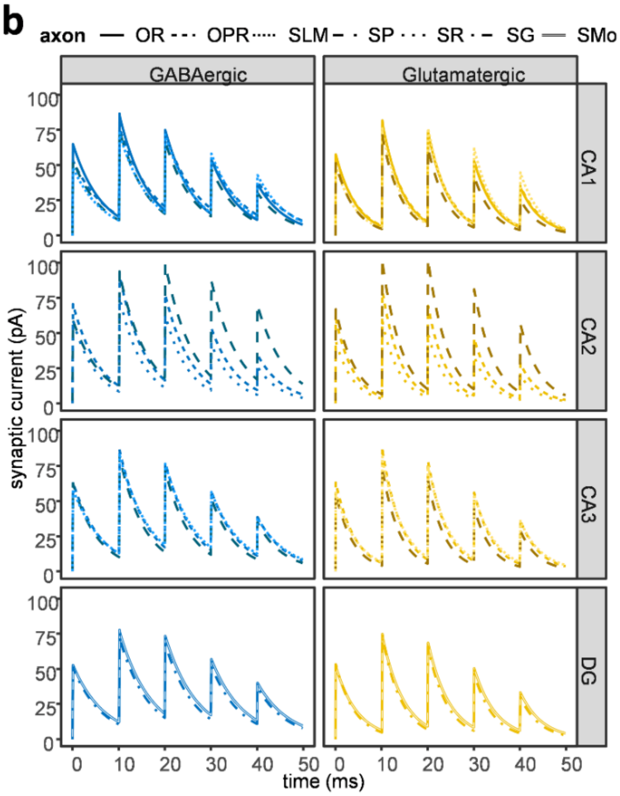

c

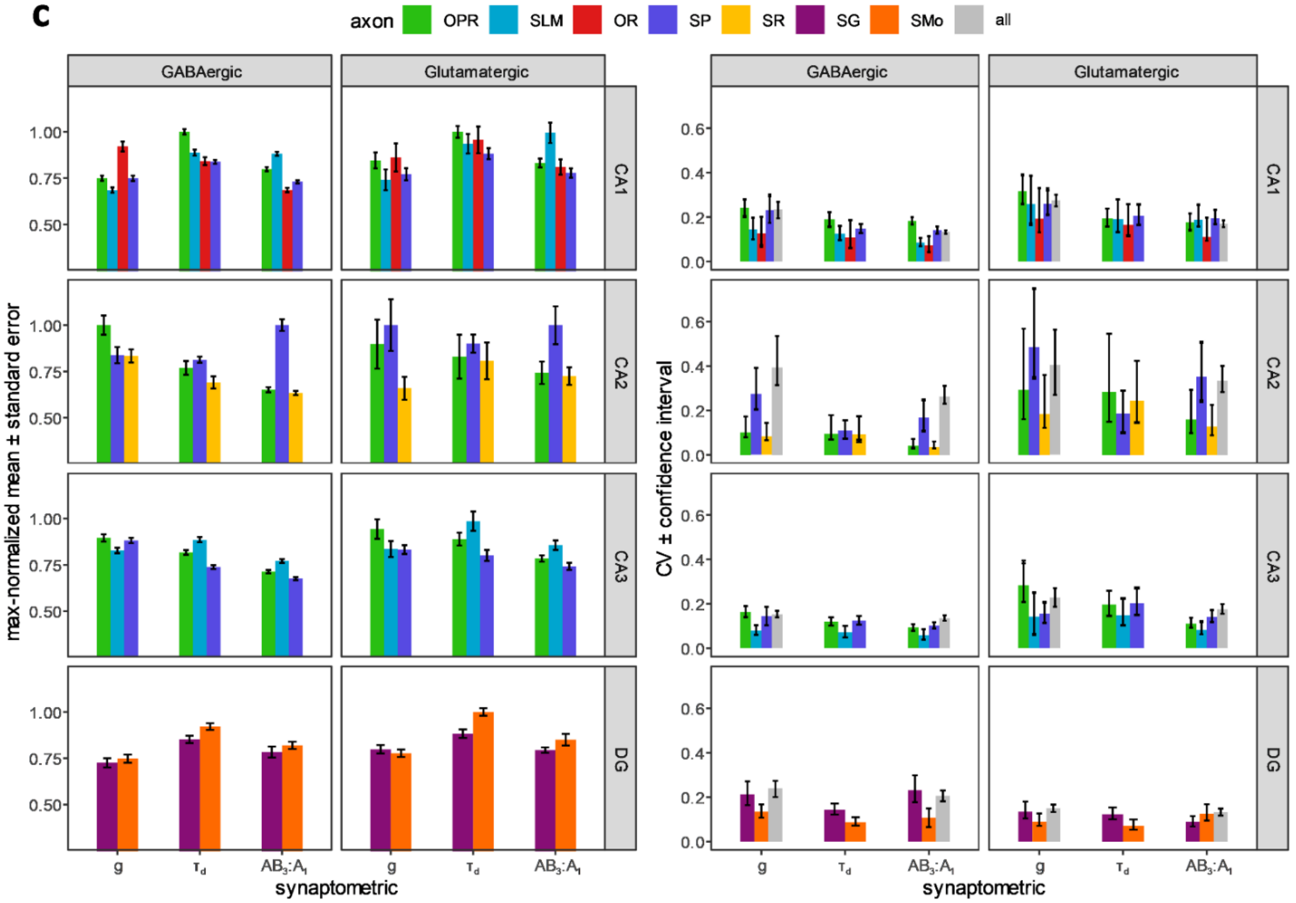

We compared synaptic properties grouped by the axonal morphology of the postsynaptic neuron: the OR group had axons in strata oriens and radiatum; OPR group was similar to OR but also had axons in stratum pyramidale; SLM group had axons in stratum lacunosum moleculare; SP group had axons only in stratum pyramidale. We also extended the study to CA2, CA3, and DG with similar grouping, if the regions had equivalent neuron types. SG and SMo in DG are homologous to SP and SLM in CA1. **(a)** We measured the average difference of synaptic parameters among groups using symmetric percentage distance (SPD). Bold values indicate statistical significance. A positive (negative) value indicates the synaptic parameter is larger (smaller) for G_1 than for G_2. **(b)** Simulated signals using averaged synaptic parameters in each group (V_h = -60 mV, E_{rev} = 0 mV, and ISI = 10 ms). **(c)** To visualize the distance of the groups **(Left)**, we normalized the trimmed-mean measures of g, τ_d, and AB_3:A_1 by dividing values to the maximum among all synapses. The error bar is the standard error. To compare the similarities within each group **(Right)**, we calculated the CV of every group and across all synapses (gray) in each region. The error bar is the confidence interval of CV. Data that is used for the analysis is available in the supplementary data.

Supplementary Figure 8: Inter-run variability of the deep learning model predictions.

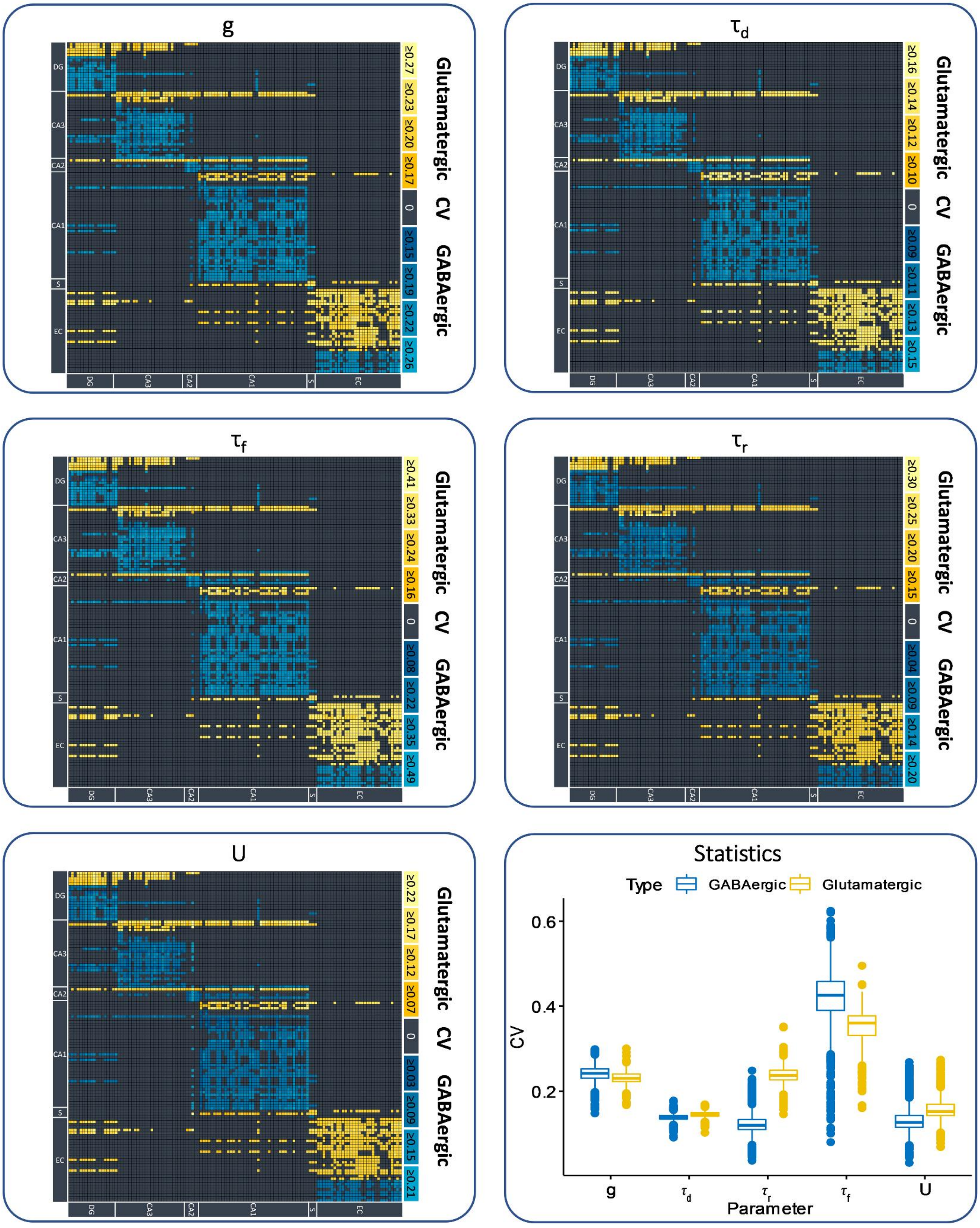

Since deep learning models depend on the (stochastic) order in which the training dataset is presented, we expected a certain degree of variation in inferences among the 100 trained models. In all analyses we reported the mean over the 100 values, but here we report the coefficient of variation (CV) of the model predictions for each synaptic parameter and potential connection. The potential connections and the synaptic parameters varied with respect to the CV. On average, τ_f had a higher CV than the other parameters.

Supplementary Figure 9: Equivalence of numerical and analytical solutions.

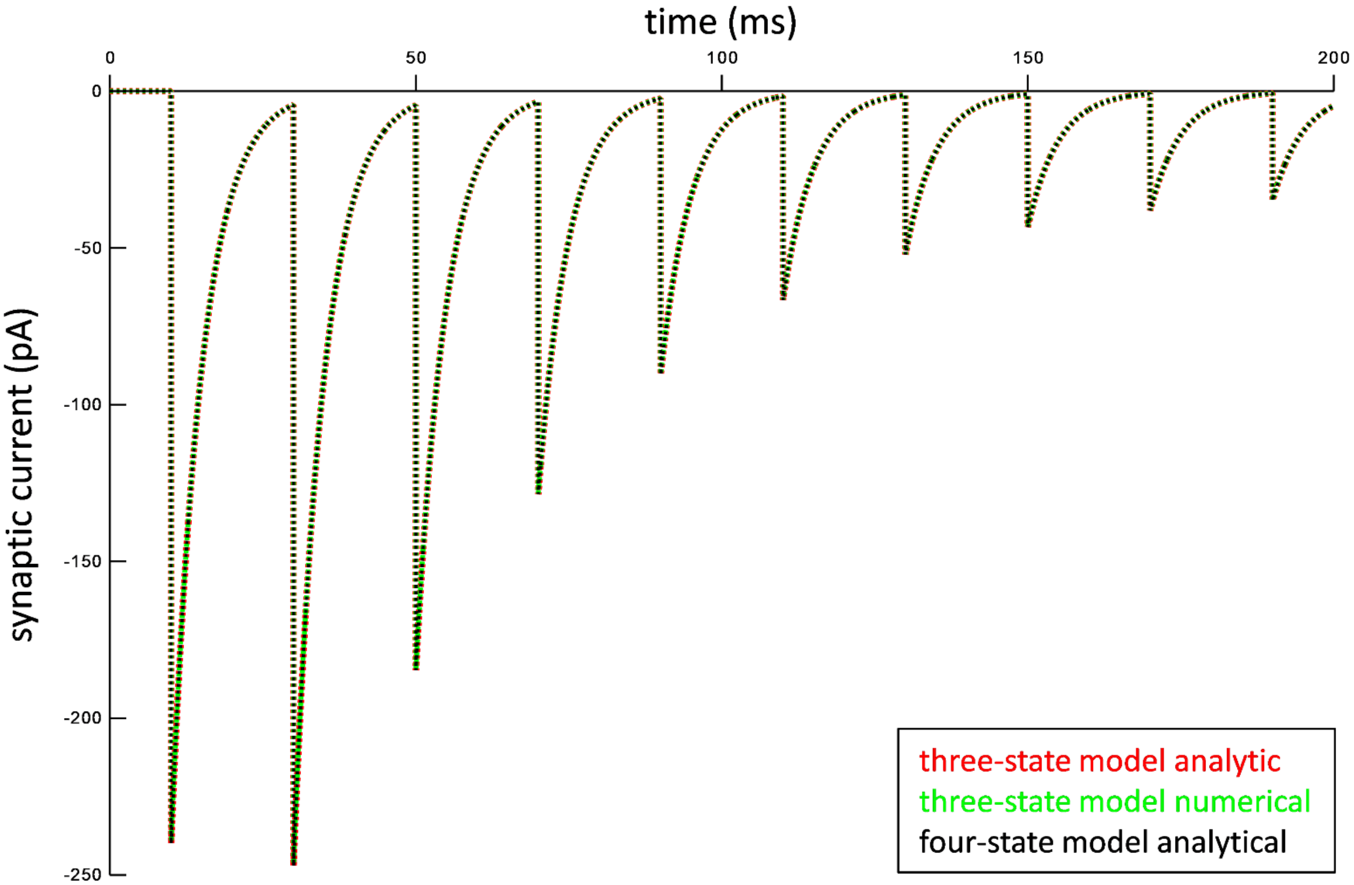

We used the NEURON simulation environment to simulate the synaptic current of a typical signal both by solving the differential equations numerically and by using the analytical equations. Moreover, we compared the results to the original ModelDB implementation of the four-state TPM model. All three simulations produced identical results, confirming the accuracy of our solution.

Supplementary Notes

Supplementary Note 1: Trace correction maximized the inclusion of studies

Background neuronal activity can change the subthreshold membrane potentials during synaptic recording. Membrane fluctuations can trigger HCN currents. Sufficient membrane depolarization can release the magnesium-block of NMDA channels. Repeated activation of GABAergic synapses may also activate $GABA_B$ receptors. Not all studies block $GABA_B$, NMDA, and HCN channels before recording synaptic signals. Fortunately, these subthreshold membrane fluctuations are slower than AMPA and $GABA_A$ signals. We thus corrected the signal for the slower membrane fluctuations before and during signal simulation to allow the inclusion of more studies using a simpler synapse model.

Supplementary Note 2: Synapse model selection

In our pipeline, we chose to simulate synaptic signals with a minimum number of data points to maximize the inclusion of studies from diverse sources. A parsimonious model also has a contained number of parameters allowing dimensionality reduction to improve machine learning performance. Tsodyks, Pawelzik, and Markram's (TPM) model only needs three data points per synaptic event to simulate signals and is fully defined by five parameters. Therefore, it was a suitable model for our study. However, experiments exclusively recording a single synaptic event only enable the estimation of g and τ_d. Traces including two successive events allow U to be also found, and with three or more events, all five constants can be computed. More synaptic events and the presence of a recovery event increase the accuracy of estimations. Of note, parameter g in our data is the product of $g_{optimization}$ times U (cf. Fig. 2b and Eq. 18). Whenever one synaptic event is available, we directly estimate g using Ohm's law and τ_d by optimization; τ_r, τ_f, and U remain missing value in the data until we infer them using the random forest model.

There are various versions of the TPM model[3, 4, 5]. A recent study investigating the dendritic impact of synaptic signals in CA1 used a complex version of the TPM model that simulates the synaptic activation time constant and the stochasticity of release[3]. Unfortunately, that version cannot analytically model the coupling of synaptic deactivation and recovery, which is required for accurate simulation of a burst of synaptic events. Therefore, we used a simplified TPM model, which also improved computational efficiency. For example, the original TPM solution has four differential equations[6, 7], but we used a subsequent formulation reducing the problem to three[6, 7]. We solved the three differential equations with the exact integration method and further simplified the resulting analytical relation to reduce the evaluations of exponential function from four to three (see Methods).

The TPM model provides a satisfactory fit for most synaptic recordings, except extremely facilitating synapses such as the giant boutons of granule cell mossy fibers in the dentate gyrus. For these synapses, the TPM model captured the phenomenology of STP, i.e., short-term facilitation, but fits were not perfect in all cases. Mossy fiber synapses had U values ranging 0.001 to 0.005, which is the smallest value possible in the Synaptic Modeling Utility. The small U means the synaptic utilization is negligible compared with a typical synapse, which has a utilization of 0.2. Small utilization values make sense in the case of DG Granule synapses, which have giant boutons and release a fraction of their resources on each synaptic event. Therefore, even in this case, the model parameters may reflect physiological aspects of synaptic signaling. A recent study suggested a revised formalism of the TPM model to address these issues[5]. It would be interesting to evaluate the performance of this model on the entire hippocampus in future studies.

Supplementary Note 3: Range and distribution of synaptic parameters are in line with their functions

The distribution of g and U were similar for GABAergic and glutamatergic synapses, suggesting optimal tuning of these synapse types to balance each other. Glutamatergic synapses had larger τ_f but smaller τ_d and τ_r values

than GABAergic synapses. Smaller τ_d means faster synaptic kinetics and therefore more precise timing with respect to signal summation and long-term plasticity. Larger τ_f means slower decay of synaptic utilization (i.e., more synaptic facilitation), and smaller τ_r, a faster recovery of synaptic resources (i.e., less synaptic depression). This pattern suggests that glutamatergic synapses are more effective in handling multiple successive events. In contrast, GABAergic synapses are more depressing and less precise in timing so as to control network activity even at the expense of resource exhaustion.

Glutamatergic τ_d values were smallest in the DG (Suppl. Fig. 6), raising the possibility that precise timing of excitatory inputs and coincidence detection may be important for this region's role in pattern separation. The range of values in the EC was limited compared with the hippocampus, suggesting a more homogeneous synaptic electrophysiology in this region. However, since the availability of synaptic data is scarce for the EC relative to the hippocampus, this latter result deserves future experimental confirmation.

Supplementary Note 4: Limited impact of temperature, membrane potential, and late postnatal development on synaptic properties

Synaptic physiology is typically studied either at room temperature or body temperature, which in rodents differ by approximately 10°C. We account for the effect of temperature when computing E_{rev} and E_j[8]. If the Q10 values of all TPM model parameters were known for all potential connections, it would be logical to normalize the temperature effect during parametric fitting. However, to the best of our knowledge such data does not exist. Consequently, we normalized temperature at the predictive modeling stage. The phenomenological impact of temperature on synaptic physiology was nominal compared to other covariates, but the change directions agreed with earlier studies, where higher recording temperature increased g and reduced τ_d[9].

Postsynaptic membrane potential (V_m) changes not only the synaptic driving force, but also the membrane time constant by activating voltage-gated ion channels like hyperpolarization-activated cyclic nucleotide-gated cation (HCN), m-type potassium, and persistent sodium (NaP) currents[10]. Therefore, even though the synaptic properties are normalized for the driving force during parametric fitting, we still included V_m in the predictive modeling stage. Our results indicate that the remaining voltage-dependent factors have less than 10% impact on synaptic parameters for a 20-mV change in V_m.

Up to twelve postnatal days, the (juvenile) brain undergoes rapid circuit construction[11]. We found the change in synaptic properties with animal age after 14 days postnatal (adolescence) is less than 10%. Therefore, adolescent animal models are acceptable templates of synaptic properties in adulthood.

Supplementary Note 5: Comparison with other studies in the literature

Our study indicates that SOM+ presynaptic neurons generate more depressing IPSCs compared to SOM- interneurons. This appears to contrast with a previous study in the neocortex (Reyes et al 1998), which reported that IPSPs generated by presynaptic SOM+ LII/III Bitufted interneurons are depressing if the postsynaptic neuron is a multipolar Pyramidal neuron, but more facilitating IPSPs if the postsynaptic neuron is another Bitufted neuron. The differences in the recorded modality may explain the discrepancy: IPSPs can be affected by the R_m and C_m of the postsynaptic neurons, rendering a direct comparison with IPSCs unfeasible. Moreover, the intracellular solution of the earlier neocortical study contained gluconate that we show might shift the synaptic ST-P properties towards facilitation. In contrast, we analyzed the synaptic properties in a more physiological condition devoid of gluconate.

Our study also suggests that glutamatergic signaling depends mostly on the presynaptic neuron type. An earlier study showed that the presynaptic group III metabotropic glutamate receptors decrease transmitter

release only at terminals contacting CA1 interneurons but not CA1 Pyramidal cells[12]. The previous report, however, relies on evoked EPSCs in organotypic slice cultures, whereas we only analyzed unitary synaptic currents systematically excluding culture conditions. It is also important to realize that many previous publications on this subject lacked proper population analysis, relying instead on exemplars.

Supplementary Note 6: Experimental validation of results

Until recently, *in vitro* multiple paired recording was the only method allowing reconstruction of recorded neurons, which is needed for identifying connected neuron types[13]. However, the advent of fast and highly sensitive voltage, calcium, and neurotransmitter indicators, along with continuous advances in microscope technology, might soon enable high-throughput gathering of synaptic signals *in vivo*. For example, the third-generation glutamate indicator iGluSnFR3 is ten times faster than its predecessors, which may allow the quantification of neurotransmitter release and reuptake dynamics[14]. The SLAP2 two-photon microscope can record synaptic activity from a full-frame window up to 18 kHz sampling rate, sufficient to record most synapses innervating a single neuron with adequate temporal resolution[15]. Light-sheet fluorescence expansion microscopy has also solved the problem of post-hoc reconstruction of circuits at the level of individual synapses[16]. Therefore, combination of these techniques may allow the direct experimental validation of synaptic physiology at the level of neuronal types in the foreseeable future.

Supplementary Note 7: Choice of synaptic data for large-scale brain simulations

Estimating synaptic parameters in voltage-clamp and current-clamp have complementary advantages and disadvantages. The voltage-clamp method provides an electrically controlled environment, enabling a straightforward calculation of g. However, the recordings may suffer from space-clamp issues, due to V_m in dendritic spines and distal dendrites not following strict somatic control[17]. Even though the current-clamp method does not have the space-clamp issue, membrane fluctuation, due to synaptic activation or background noise, can activate subthreshold voltage-gated ion channels[10]. In distal dendrites, due to passive normalization, the local dendritic synaptic potential may be sufficient to activate ion channels involved in spike generation[17].

We reported just the somatic impact of synaptic inputs. The normalized values produced in the present work, together with the existing number of contacts and connection probabilities[18], which are available on Hippocampome.org, could thus be used in conjunction with a more comprehensive model containing digitally reconstructed neuronal morphologies to infer the biophysical properties of individual synapses locally on dendrites. It is possible to derive the dendritic synaptic parameters from somatic recordings using the morphological reconstruction of the postsynaptic neuron and a detailed account of all subthreshold-voltage-gated ionic channels[3, 19]. Yet, in most cases, such data are not available. Nevertheless, the estimation of g and τ_d using current-clamp should be closer to the local dendritic events since even a simple RC neuron model, as used in this study, can reduce the degree of dendritic filtering. Therefore, we suggest that synaptic inferences in current-clamp are suitable for network simulations that use complex neuron models incorporating the dendrites. Voltage-clamp, in contrast, better estimates the somatic impact of synaptic events since it includes dendritic filtering. Thus, we recommend use of voltage-clamp synaptic estimates for network simulations that use point-neuron models.

Supplementary References

1. Guzman SJ, Schlogl A, Frotscher M, Jonas P. Synaptic mechanisms of pattern completion in the hippocampal CA3 network. *Science* **353**, 1117-1123 (2016).

2. McBain CJ, DiChiara TJ, Kauer JA. Activation of metabotropic glutamate receptors differentially affects two classes of hippocampal interneurons and potentiates excitatory synaptic transmission. *The Journal of neuroscience : the official journal of the Society for Neuroscience* **14**, 4433-4445 (1994).

3. Ecker A*, et al.* Data-driven integration of hippocampal CA1 synaptic physiology in silico. *Hippocampus* **30**, 1129-1145 (2020).

4. Zucker RS, Regehr WG. Short-term synaptic plasticity. *Annual review of physiology* **64**, 355-405 (2002).

5. Rossbroich J, Trotter D, Beninger J, Toth K, Naud R. Linear-nonlinear cascades capture synaptic dynamics. *PLoS Comput Biol* **17**, e1008013 (2021).

6. Morrison A, Diesmann M, Gerstner W. Phenomenological models of synaptic plasticity based on spike timing. *Biological cybernetics* **98**, 459-478 (2008).

7. Tsodyks M, Pawelzik K, Markram H. Neural networks with dynamic synapses. *Neural Comput* **10**, 821-835 (1998).

8. Moradi K, Ascoli GA. A comprehensive knowledge base of synaptic electrophysiology in the rodent hippocampal formation. *Hippocampus* **30**, 314-331 (2020).

9. Otis TS, Mody I. Modulation of decay kinetics and frequency of GABAA receptor-mediated spontaneous inhibitory postsynaptic currents in hippocampal neurons. *Neuroscience* **49**, 13-32 (1992).

10. Moradi K, Kaka G, Gharibzadeh S. The role of passive normalization, voltage-gated channels and synaptic scaling in site-independence of somatic EPSP amplitude in CA1 pyramidal neurons. *Neurosci Res* **73**, 8-16 (2012).

11. Isshiki M, Tanaka S, Kuriu T, Tabuchi K, Takumi T, Okabe S. Enhanced synapse remodelling as a common phenotype in mouse models of autism. *Nat Commun* **5**, 4742 (2014).

12. Scanziani M, Gahwiler BH, Charpak S. Target cell-specific modulation of transmitter release at terminals from a single axon. *Proc Natl Acad Sci U S A* **95**, 12004-12009 (1998).

13. Moradi K, Ascoli GA. Systematic data mining of hippocampal synaptic properties. In: *Hippocampal Microcircuits A Computational Modeler's Resource Book* (ed^(eds Cutsuridis V, Graham BP, Cobb S, Vida I). 2 edn. Springer International Publishing (2019).

14. Marvin JS*, et al.* Stability, affinity, and chromatic variants of the glutamate sensor iGluSnFR. *Nature methods* **15**, 936-939 (2018).

15. Podgorski K. Advances in two-photon projection microscopy for high-speed brain imaging. In: *High-Speed Biomedical Imaging and Spectroscopy VI* (ed^(eds). International Society for Optics and Photonics (2021).

16. Burgers J*, et al.* Light-sheet fluorescence expansion microscopy: fast mapping of neural circuits at super resolution. *Neurophotonics* **6**, 015005 (2019).

17. Beaulieu-Laroche L, Harnett MT. Dendritic Spines Prevent Synaptic Voltage Clamp. *Neuron* **97**, 75-82 e73 (2018).

18. Tecuatl C, Wheeler DW, Ascoli GA. A Method for Estimating the Potential Synaptic Connections Between Axons and Dendrites From 2D Neuronal Images. *Bio Protoc* **11**, e4073 (2021).

19. Baker JL, Perez-Rosello T, Migliore M, Barrionuevo G, Ascoli GA. A computer model of unitary responses from associational/commissural and perforant path synapses in hippocampal CA3 pyramidal cells. *Journal of computational neuroscience* **31**, 137-158 (2011).
