# Supplementary material for: Normalized unitary synaptic signaling of the hippocampus and entorhinal cortex predicted by deep learning of experimental recordings
Source: Commun Biol. 2022 May 5;5:418. doi: 10.1038/s42003-022-03329-5 (PMC9072429; doi:10.1038/s42003-022-03329-5)
Supplement: Supplementary file 2 — Description of Additional Supplementary Files [file 42003_2022_3329_MOESM2_ESM.pdf]

Description of Additional Supplementary Files

File name: Supplementary Data 1

Description: Data underlying Fig. 6a, Fig. 8, and Suppl. Fig. 7; and p-values for Fig. 8b.

File name: Supplementary Data 2

Description: List of all the papers leveraged in the current study.

File name: Supplementary Movie 1

Description: Synapses with smaller and larger amplitudes tend to undergo short-term facilitation and depression, respectively. We sorted all entorhinal-hippocampal synapses by conductance (high-amplitude to low-amplitude) and simulated each synapse separately in voltage-clamp and standard condition with ISI = 20 ms. We animated the evolution of synaptic currents as a function of conductance, which revealed the transition from depression to facilitation.
